# Social determinants of health in relation to firearm-related homicides in the United States: A nationwide multilevel cross-sectional study

**DOI:** 10.1371/journal.pmed.1002978

**Published:** 2019-12-17

**Authors:** Daniel Kim

**Affiliations:** Department of Health Sciences, Bouvé College of Health Sciences, Northeastern University, Boston, Massachusetts, United States of America; Barts and the London School of Medicine & Dentistry Queen Mary University of London, UNITED KINGDOM

## Abstract

**Background:**

Gun violence has shortened the average life expectancy of Americans, and better knowledge about the root causes of gun violence is crucial to its prevention. While some empirical evidence exists regarding the impacts of social and economic factors on violence and firearm homicide rates, to the author’s knowledge, there has yet to be a comprehensive and comparative lagged, multilevel investigation of major social determinants of health in relation to firearm homicides and mass shootings.

**Methods and findings:**

This study used negative binomial regression models and geolocated gun homicide incident data from January 1, 2015, to December 31, 2015, to explore and compare the independent associations of key state-, county-, and neighborhood-level social determinants of health—social mobility, social capital, income inequality, racial and economic segregation, and social spending—with neighborhood firearm-related homicides and mass shootings in the United States, accounting for relevant state firearm laws and a variety of state, county, and neighborhood (census tract [CT]) characteristics. Latitude and longitude coordinates on firearm-related deaths were previously collected by the Gun Violence Archive, and then linked by the British newspaper *The Guardian* to CTs according to 2010 Census geographies. The study population consisted of all 74,134 CTs as defined for the 2010 Census in the 48 states of the contiguous US. The final sample spanned 70,579 CTs, containing an estimated 314,247,908 individuals, or 98% of the total US population in 2015. The analyses were based on 13,060 firearm-related deaths in 2015, with 11,244 non-mass shootings taking place in 8,673 CTs and 141 mass shootings occurring in 138 CTs. For area-level social determinants, lag periods of 3 to 17 years were examined based on existing theory, empirical evidence, and data availability. County-level institutional social capital (levels of trust in institutions), social mobility, income inequality, and public welfare spending exhibited robust relationships with CT-level gun homicide rates and the total numbers of combined non-mass and mass shooting homicide incidents and non-mass shooting homicide incidents alone. A 1–standard deviation (SD) increase in institutional social capital was linked to a 19% reduction in the homicide rate (incidence rate ratio [IRR] = 0.81, 95% CI 0.73–0.91, *p <* 0.001) and a 17% decrease in the number of firearm homicide incidents (IRR = 0.83, 95% CI 0.73–0.95, *p* = 0.01). Upward social mobility was related to a 25% reduction in the gun homicide rate (IRR = 0.75, 95% CI 0.66–0.86, *p <* 0.001) and a 24% decrease in the number of homicide incidents (IRR = 0.76, 95% CI 0.67–0.87, *p <* 0.001). Meanwhile, 1-SD increases in the neighborhood percentages of residents in poverty and males living alone were associated with 26%–27% and 12% higher homicide rates, respectively. Study limitations include possible residual confounding by factors at the individual/household level, and lack of disaggregation of gun homicide data by gender and race/ethnicity.

**Conclusions:**

This study finds that the rich–poor gap, level of citizens’ trust in institutions, economic opportunity, and public welfare spending are all related to firearm homicide rates in the US. Further establishing the causal nature of these associations and modifying these social determinants may help to address the growing gun violence epidemic and reverse recent life expectancy declines among Americans.

## Abstract 2

Daniel Kim uses geolocated gun homicide data to investigate associations between local social determinants of health and firearm deaths.

## Author summary

**Why was this study done?:**

- Better knowledge about the root causes of gun violence is crucial to its prevention.
- While some empirical evidence exists regarding the impacts of social and economic factors on violence and firearm homicide rates, to the author’s knowledge, there has yet to be a comprehensive and comparative lagged, multilevel investigation of major social determinants of health in relation to gun homicides and mass shootings.

**What did the researchers do and find?:**

- This study used geolocated gun homicide incident data from the US in 2015 to explore the independent associations of key state-, county-, and neighborhood-level social determinants of health—social mobility, social capital, income inequality, residential racial and economic segregation, and social spending—with neighborhood firearm homicides and mass shootings in the US.
- The study population consisted of all 74,134 census tracts (CTs) as defined for the 2010 Census in the 48 states of the contiguous US.
- Analyses were based on 13,060 firearm-related deaths in 2015, with 11,244 non-mass shootings taking place in 8,673 CTs and 141 mass shootings occurring in 138 CTs.
- Using lag periods of 3 to 17 years, county-level institutional social capital, social mobility, income inequality, and public welfare spending showed robust relationships with CT-level gun homicide rates and the numbers of combined fatal non-mass and mass shooting incidents and non-mass shooting incidents alone.

**What do these findings mean?:**

- This study finds that the rich–poor gap, levels of citizens’ trust in institutions, economic opportunity, and public welfare spending are all associated with firearm-related homicide rates and incident counts in the US.
- Establishing the causal nature of these relationships and modifying these social determinants may help to combat the ongoing gun violence epidemic and aid in reversing recent life expectancy declines among Americans.

## Introduction

The US is grappling with a worsening epidemic of gun homicides. Between 2001 and 2013, guns took the lives of more Americans than the total number killed by war, AIDS, illegal drug overdoses, and terrorism combined during the same period [1]. Gun homicides rose consecutively in 2015, 2016, and 2017 [2], and are the second leading cause of injury death among youths and young adults [3].

Identifying the key drivers of firearm violence is crucial to its prevention. While a growing literature has explored the impacts of state gun control policies [4–7], there are increasing calls for comprehensive, multidisciplinary approaches to unpack and address the root causes—the social determinants—of gun homicides [7–9]. As described by the World Health Organization, the social determinants of health are the upstream social and economic conditions in which people are born, grow, live, work, and age that shape individual as well as group differences in health status [10]. These social determinants of health include social capital, reflecting informal and formal social ties within society; income inequality, the divide between the rich and poor; residential racial segregation and economic segregation, which correspond to the physical separation of 2 or more groups as defined by race/ethnicity and socioeconomic status, respectively, into different neighborhoods; non-medical social spending such as government spending on welfare and education; and intergenerational social mobility—the ability of children to climb higher on the social ladder than their parents [11,12].

Overall, the empirical literature linking area-level social determinants of health to homicides is limited by factors including the study designs used (e.g., primarily ecological and cross-sectional rather than multilevel and lagged/longitudinal designs), studies’ controlling for only a limited number of covariates (with a greater possibility of residual confounding), and studies’ lack of specificity to firearm-related homicides. Investigations on social mobility are also sparse. Furthermore, results from cross-sectional studies that do not employ time lags are problematic due to the possibility of reverse causation; for example, higher levels of violent crime have been linked to lower levels of social trust in neighbors in the subsequent year [13], such that the direction of the association between social trust and violent crime is less clear if temporal order is not taken into account. Even in lagged cross-sectional or longitudinal studies of social determinants and homicide, the lack of exploration of varying lag periods to identify the most sensitive lag periods is a critical empirical gap.

Notably, studies so far have focused analytically on single social determinants, which can introduce confounding by other social determinants at the same and other geographic levels, and can hamper our ability to directly compare the relative sizes of impacts across social determinants. To date, no studies to the author’s knowledge have comprehensively investigated and compared major social determinants of health as drivers of firearm homicides and mass shootings. To address this gap and to capitalize on the recent public release of geolocation data on reported gun homicides for the year 2015 in the US, this study explores and compares the independent associations of state-, county-, and neighborhood-level income inequality, racial and economic segregation, social spending, social capital, and social mobility with neighborhood firearm homicides and mass shootings, after taking into account relevant state firearm laws and state-, county-, and neighborhood-level characteristics. In contrast to previous studies, this study directly compares the magnitude of associations across multiple social determinants in the same models. This study further explores associations using multiple lag periods for 2 key social determinants, social spending and income inequality, to identify the most sensitive lag periods.

## Methods

All study analyses were planned (without a formal prospective analysis plan document) prior to undertaking this lagged multilevel cross-sectional study, with the exception of the analysis of additional lag periods for social spending and income inequality, as noted in the statistical analysis section below.

### Study population

The study population consisted of all 74,134 census tracts (CTs) as defined for the 2010 Census in the 48 states of the contiguous US. CTs are geographic entities within counties (or the statistical equivalent of counties) that generally vary in population size from 1,200 to 8,000 residents.

### Outcomes

Primary outcomes were the 2015 firearm-related homicide rate and the total number of firearm homicide incidents, non-mass shooting homicide incidents, and mass shooting homicide incidents, all measured at the CT level. Latitude and longitude coordinates on firearm-related homicides were previously collected by the Gun Violence Archive (GVA), a non-profit organization that tracks shootings and gun deaths in the US using media reports [14]. The GVA uses automated queries, and manually searches over 2,000 media sources, aggregates, police blotters, police media outlets, and other sources daily. Each incident is verified by both initial researchers and secondary validation processes [14]. The geolocated gun homicide data do not include information to allow for disaggregating homicides by victim and perpetrator characteristics including race and gender, and whether the homicides were perpetrated by law enforcement versus other individuals. In 2017, the British newspaper *The Guardian* linked each of the geolocated gun murders in 2015 to a CT according to 2010 Census geographies and publicly released the data [15].

For each CT, the homicide rate was calculated as the total number of reported firearm homicides divided by the total population in 2015. Non-mass shooting homicide and mass shooting homicide incidents were defined as the total number of reported incidents in the CT in 2015 in which an individual used a firearm to kill 1–2 and 3 or more persons, respectively, not including the shooter. This definition of a mass shooting comes from the Investigative Assistance for Violent Crimes Act of 2012 signed into law by the US Congress in 2013 [16].

For the analysis of non-mass shooting homicide incidents, 138 CTs with at least 1 mass shooting incident were excluded from the sample. For the analysis of mass shooting homicides, incidents with 1–2 deaths were not included.

For area-level social determinants, average lag periods of 3 years up to a maximum of 17 years were examined. With the exception of income inequality, for which evidence points to lag periods on the order of a decade or longer [17,18], and residential segregation, for which available data were for the year 2000, year-specific social determinant data corresponding to shorter lag periods (a decade or less) were selected, to better reflect plausible lag periods for hypothesized effects. A more detailed description of the primary evidence on and the rationale for the choice of lag periods used in the main models is provided in S2 Table [19–37]. For state and local (county and municipal) social spending and county-level income inequality, for which data across multiple years were available, lag periods of 5 and 7 years, respectively, were originally used. In response to the peer review process, additional average lag periods of 3, 7, and 10 years for social spending and 3 and 17 years for income inequality were investigated (using unitary changes of $250 per capita for social spending and a 0.1-Gini unit increase for income inequality), given that the most sensitive lag periods have yet to be established (S2 Table). Models using 3-year lags for social spending with otherwise the same model specifications did not converge. Estimates according to the 5-year average lag for social spending and the 17-year average lag for income inequality exhibited the most consistent and smallest *p*-values, signifying a lower likelihood of being observed due to chance (see S3 and S4 Tables). These lag periods were hence used for the main analyses.

### County- and commuting zone-level social determinant exposures

County-level intergenerational social mobility was based on Chetty and colleagues’ measure of absolute upward income mobility, which they have described as an indicator of economic opportunity [11]. This measure was constructed using 2010–2012 income tax return data on over 10 million individuals born in 1980–1982 whose parents could be identified through tax return data and whose mean parent income between 1996 and 2000 was strictly positive (thereby excluding 1.2% of children). Counties were assigned on the basis of the child’s zip code of residence at the age of 15 years (1995–1997). For a given county, this measure signifies the average income rank that individuals born to the poorest quartile of parents were able to attain. Higher average income ranks reflect greater intergenerational social mobility [11].

County-level income inequality was measured using the Gini coefficient derived from household income reported in the 2006–2010 and 2010–2014 American Community Survey (ACS) [19] and the Internal Revenue Service Statistics of Income sample [20]. County-level Gini coefficients estimated using the latter data were calculated by Chetty and colleagues for mean family income in 1996–2000 according to federal income tax records for the parents included in their core sample used to calculate social mobility [11]. The Gini coefficient can assume theoretical values ranging from 0 (perfect equality) to 1 (perfect inequality).

Measures of residential segregation according to race/ethnicity and income were constructed by Chetty and colleagues using the 2000 Census [11]. Racial segregation was measured using Theil’s entropy index, which captures the extent to which the racial distribution in each CT deviates from the overall racial distribution in the commuting zone (CZ), an aggregate of counties meant to spatially represent the local labor market. Following Iceland [38], Chetty et al. [11] calculated *H*, the degree of racial segregation in the CZ, using the formula

$$H=\sum_{j}\left[\frac{{\text{pop}}_{j}}{{\text{pop}}_{\text{total}}}\times \frac{E-Ej}{E}\right]$$

where pop_*j*_ indicates the total population of CT *j* and pop_total_ denotes the total population of the CZ; *E* is an entropy index that measures the level of racial diversity in the CZ; *j* indexes CTs in the CZ; and *E*_*j*_ denotes the level of racial diversity in tract *j*. *H* takes on a theoretical value of 1 when there is no racial heterogeneity within CTs (corresponding to complete segregation)—since *E*_*j*_ = 0 in all tracts—and a value of 0 when all tracts have the same racial composition as the CZ as a whole, since *E*_*j*_ = *E* [11].

Following Reardon and Firebaugh [39], Chetty et al. [11] estimated the degree to which individuals below the *x*th percentile of the local household income distribution were segregated from individuals above the *x*th percentile in each CZ. For each CZ, they then calculated income segregation as the weighted average of income segregation at each percentile, with greater weight placed on percentiles in the middle of the income distribution.

Social capital was measured at the county level using 2 validated indices, a community social capital index and an institutional social capital index [24]. The community social capital index was designed to measure levels of both informal and formal civic engagement/participation at the community level, while the institutional social capital index was developed to capture levels of trust and confidence in institutions that extend beyond the community and include the government, the media, and corporations.

To construct these county-level social capital indices, the developers first created state-level indices. Principal components analysis (PCA) was used to identify different domains of social capital [24], and then both content validity and internal consistency reliability were employed to delineate the items to include in the state-level community and institutional social capital indices. The state community and institutional social capital indices had Cronbach’s α values of 0.92 and 0.72, respectively [24]. Because indicators of informal civic engagement at the county level were unavailable, the developers created an index of informal civic engagement at the state level using available data from the Current Population Survey (CPS). The index score was the first principal component score combining 6 items. This index score was then assigned to each county within a state. Next, at the county level, the developers created 5 different candidate indices, using various combinations of the informal civic engagement index score and the numbers of county-level membership organizations per capita, non-religious non-profit organizations per capita, and congregations per capita. These indices were estimated using PCA. Finally, for each of the candidate indices, the developers computed the population-weighted average index score across a state’s counties, and calculated the correlation between each of the state averages and the state-level community social capital index. Out of the candidate indices, the index with the strongest correlation was selected [24].

A similar process was undertaken by the developers for the county-level institutional social capital index due to the lack of data at the county level on confidence in institutions. At the state level, an institutional confidence index was first created that included 3 institutional confidence variables [24]. Three versions of a county-level institutional social capital index were then developed using different combinations of presidential voting rate, census response rate, and the confidence index. The developers next calculated population-weighted state averages across a state’s counties and compared the correlations with the state-level institutional social capital index to determine the items in the final index [24].

Estimated correlations with demographic, socioeconomic, and health factors that measure criterion and construct validity indicated that the county-level community and institutional social capital indices performed comparably to or even better than the Penn State social capital index, another US county-level social capital index [24]. The community and institutional social capital indices also improved upon the Penn State social capital index by including indicators tapping into additional concepts related to social capital such as volunteerism and informal community engagement [24].

The county-level community social capital index combined the number of membership organizations per capita, the number of non-profit (religious and non-religious) organizations per capita, and the percentages of adults who reported in the past year volunteering for an organization, attending a public meeting to discuss community affairs, working with neighbors to improve the community, serving on a committee or as an officer of a group, attending a public meeting where political issues were discussed, and participating in a march, protest, rally, or demonstration. Data sources consisted of the 2008 and 2013 Civic Engagement Supplements to the CPS and the 2015 Volunteer Supplement to the CPS [24], the 2015 Internal Revenue Service Business Master File [26], and the 2010 Religious Congregations and Membership Study [24].

The county-level institutional social capital index was derived from the average rate at which citizen adults of voting age cast ballots in the 2012 and 2016 presidential elections, the response rate for residents returning the 2010 decennial census questionnaire through the mail, and the percentage of adults with “great” or “some” confidence in institutions (corporations, the media, and public schools) “to do what is right”. These data came from US Election Assistance Commission annual reports [27,28], the Census Bureau, and the 2013 Civic Engagement Supplement to the CPS [25].

### State and local social determinant exposures

State and local government social spending (public welfare, education, protection, and total per capita) corresponded to the 2005, 2008, 2010, and 2012 fiscal years as reported by the Annual Survey of State and Local Government Finances [29]. Welfare spending encompasses state supplements for unemployment insurance, workers’ compensation, work incentive programs, public assistance programs (e.g., Aid to Families with Dependent Children), and state supplements for the Supplemental Security Income (SSI) program for the aged, blind, and disabled. Education spending consists of local spending on elementary and secondary school education and financial aid to college students, with higher spending conceivably reducing the financial burden of parents. Protection spending reflects spending on state and local police, corrections/prisons, and local fire protection.

S2 Table provides a list of the key social determinants examined, their data sources, and their average lag periods in relation to the outcomes, both in the current study and in previous studies. Additional details on how lag periods were selected are given within and beneath the table.

### Covariates

All models controlled for multiple CT-, county-, CZ-, and state-level characteristics. At all geographic levels, median household income and the percentage of black individuals were covariates, since they were considered key potential confounders, particularly for social determinants (e.g., social mobility) measured at the same geographic level. For example, all models controlled for median household income at the county level. This should have had the effect of reducing confounding by county-level socioeconomic status of the associations between the social capital indices and gun homicides. CT-level demographic and socioeconomic covariates included median household income, percentage with high school education, percentage black, percentage male, percentage unemployed, percentage receiving cash assistance, percentage in poverty, percentage age 20–34 years, percentage of males living alone, and total population centered in the year 2012 (based on 5-year estimates from the 2010–2014 ACS). Many of these covariates have been found to be related to violent crime or have been included as control variables in other homicide studies [5,40–43]. At the county level, covariates included median household income and the percentage black (5-year averages based on the 2006–2010 ACS), and population density and property crime rates, based on Area Resource File data obtained through the Inter-university Consortium for Political and Social Research archive [44]. At the CZ level, models were adjusted for median household income, the percentage black, and a dichotomous variable to indicate whether the CZ corresponded to an urban area. State-level covariates included total state and local social spending per capita and state gun control policy indicators for concealed weapon carry laws, requirements for gun dealers to report records to the state, and state background check laws—with each of these indicators being shown in previous studies to be associated with firearm-related homicide rates [4–6]. State fixed effects or fixed effects for the 9 census statistical divisions were further included to control for area-level socioeconomic and institutional factors that might be jointly correlated with the social determinants examined and firearm-related homicides.

### Statistical analysis

Due to statistical evidence of observed variance greater than expected, negative binomial models (variants of Poisson regression models) and zero-inflated negative binomial models were considered for all analyses. Zero-inflated negative binomial regression is used for modeling count variables with excessive zero counts, whereby excess zero counts are in theory generated by a separate process from the count values and so the excess zeros are modeled independently [45]. Negative binomial models and zero-inflated negative binomial models that omitted state fixed effects were used to permit convergence of the zero-inflated negative binomial models and to enable comparable model covariates in the 2 models.

The Vuong test [46] and Clarke sign test [47] are 2 statistical tests that can be used to choose between models based on model fit. These tests yielded inconsistent results: the positive value of the Vuong test statistic (Z = 6.69, *p <* 0.001) indicated that the zero-inflated negative binomial model was closer to the true model, but the negative value of the Clarke sign statistic (M = −3,161.50, *p <* 0.001) signified that the negative binomial model was the preferred model. Due to the inconsistency of the results from the Vuong and Clarke sign tests with the same null hypothesis, the negative binomial model was selected for the main analyses in order to permit state fixed effects to be included in all models to minimize confounding.

For homicide rate, the negative binomial models used the number of firearm-related homicides in a CT in 2015 as the outcome and the log of the CT population as the offset. For the number of homicide incidents (total, non-mass shooting, and mass shooting), models employed the total number of corresponding incidents in a CT in 2015 as the dependent variable. All models applied the log link function, by exponentiating the exposure variables to link them to the outcome. For each outcome, hierarchical multivariate-adjusted negative binomial regression models were estimated as follows, controlling for all of the above covariates at the state, CZ, county, and CT levels:

**Model 1**: Income inequality + social spending (welfare, education, protection) + racial segregation
**Model 2**: Model 1 + income segregation
**Model 3**: Model 2 + social capital (community, institutional)
**Model 4**: Model 3 + social mobility

This hierarchical modeling process enabled an investigation of the independent impacts of social determinants, and consideration of possible mediation for the income inequality, social spending, and racial segregation effects by income segregation, social capital, and social mobility.

To reduce the chances of mis-specification of the models due to nonlinear associations, social determinant exposure variables and covariates were first examined using polynomial (quadratic) terms and log transformation and model goodness-of-fit statistics. Where there was statistical evidence of a nonlinear association and a better model fit, the corresponding transformation was used. Model goodness-of-fit was assessed using the quasi-Akaike information criterion QIC and QICu statistics and the %QIC SAS macro proposed by Pan [48] for generalized estimating equation (GEE) models. A smaller difference between the QIC and QICu statistics indicated a better model fit [48].

All continuous social determinant exposure variables were standardized to a 1–standard deviation (SD) change to allow for direct comparisons of effect sizes across social determinants. All associations are reported as incidence rate ratios (IRRs) and their 95% confidence intervals (CIs). All variance inflation factors (VIFs) for primary social determinants had values less than 5, and were hence considered to be in the acceptable range (S5 Table) [49].

Attenuation of the estimate and an increase in the *p*-value corresponding to the association for income inequality, welfare spending, or racial segregation in Model 1 after controlling for income segregation, social capital, or social mobility in a subsequent model would satisfy 2 of the 3 standard steps to test for potential mediation according to the Baron and Kenny mediation analysis approach [50]. In such instances, a subsequent model was examined to determine whether the Gini coefficient, welfare spending, or racial segregation was associated with the respective potential mediator, i.e., to evaluate the remaining step of the Baron and Kenny approach [50]. The correct temporal order (i.e., with the social determinant year of measurement preceding the mediator year of measurement, in turn preceding the homicide outcome year of 2015) was satisfied for 2 of these 3 mediation analyses. The one exception was income segregation as a mediator of racial segregation, for which data were available for both factors only from the 2000 Census (see S2 Table). Causal mediation analyses using the approach described by Valeri and VanderWeele [51] were also undertaken to estimate the percentages of the total effect due to direct, indirect, and interaction effects, while incorporating exposure–mediator interaction terms. If no such interaction is present, then the direct and indirect effects are equivalent between this approach and the Baron and Kenny approach [50].

In sensitivity analyses, CTs that represented extreme outliers for homicide rates (>40 homicides/1,000 persons) were excluded. To match the Federal Bureau of Investigation’s definition of a mass shooting as 4 or more people killed in a single incident, not including the shooter [52], an alternative definition of non-mass shootings in which the shooter used a firearm to kill up to 3 persons was further adopted. The robustness of the findings when protection spending was limited to police protection services only (i.e., excluding spending on fire protection and corrections/prisons) and when the following factors were added individually to the models was also assessed: percentage Hispanic, percentage Asian and Pacific Islander, percentage non-Hispanic white, percentage 18 years of age and younger, percentage 65 years of age and older, and percentage of children in poverty. Finally, because of past empirical associations of state-level income inequality and other health outcomes, the Gini coefficient was examined at the state level (using 2000 Census data) instead of at the county level.

All standard errors took into account the similarity (clustering) of CT-level outcomes within the same state using a GEE approach [53]. All analyses were conducted using SAS software, version 9.4. Since all study data were in the public domain, this study was approved for an exemption from human subjects by the Office of Human Subject Research Protection at Northeastern University.

## Results

**Fig 1:**
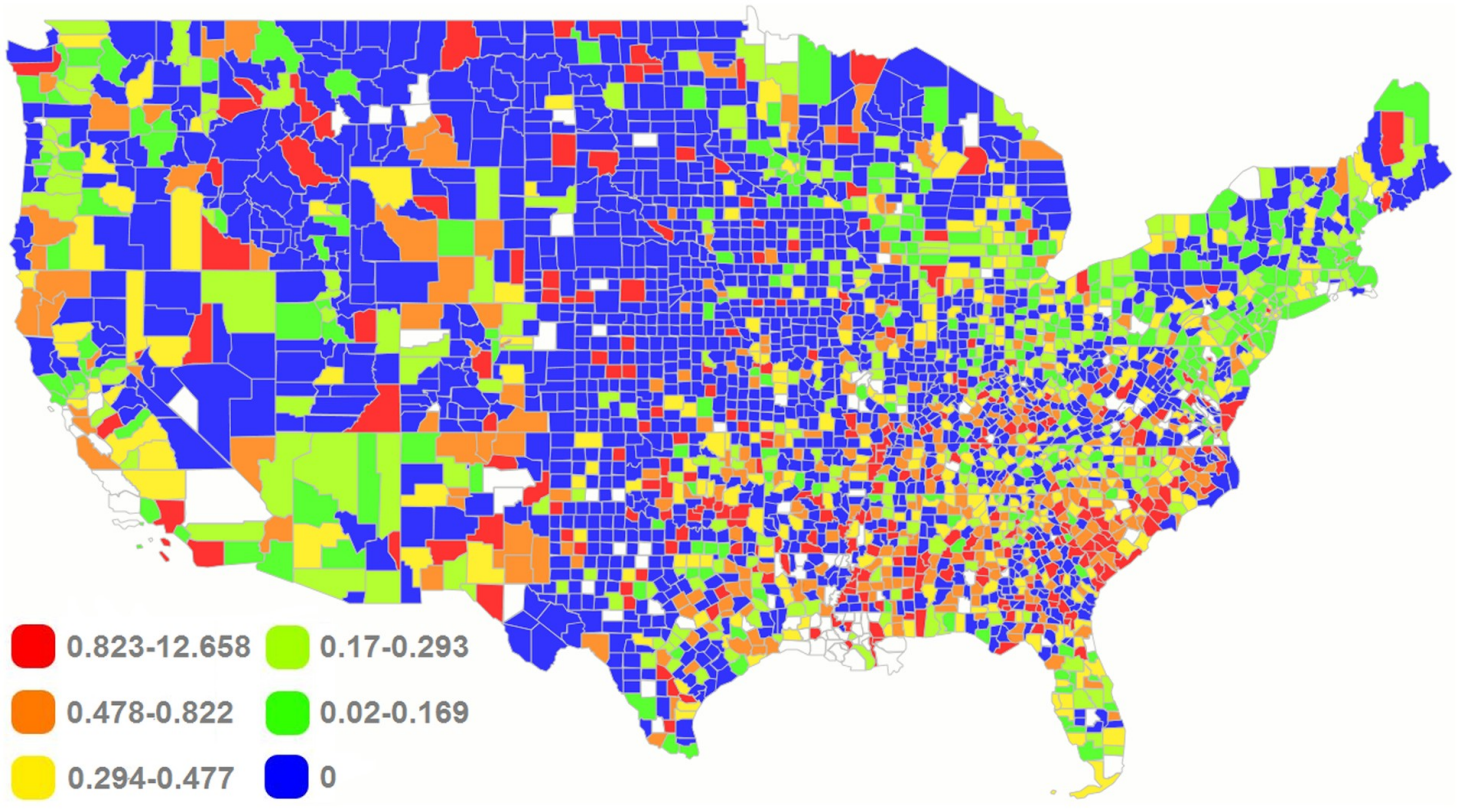
Heat map of gun homicide rate per 10,000 population by county in the contiguous US, 2015. US counties with positive homicide rates are color-coded into quintiles. White indicates counties for which no data were available. Created with Mapline mapping software (https://mapline.com), which uses OpenStreetMap geodata (https://openstreetmap.org).

**Fig 2:**
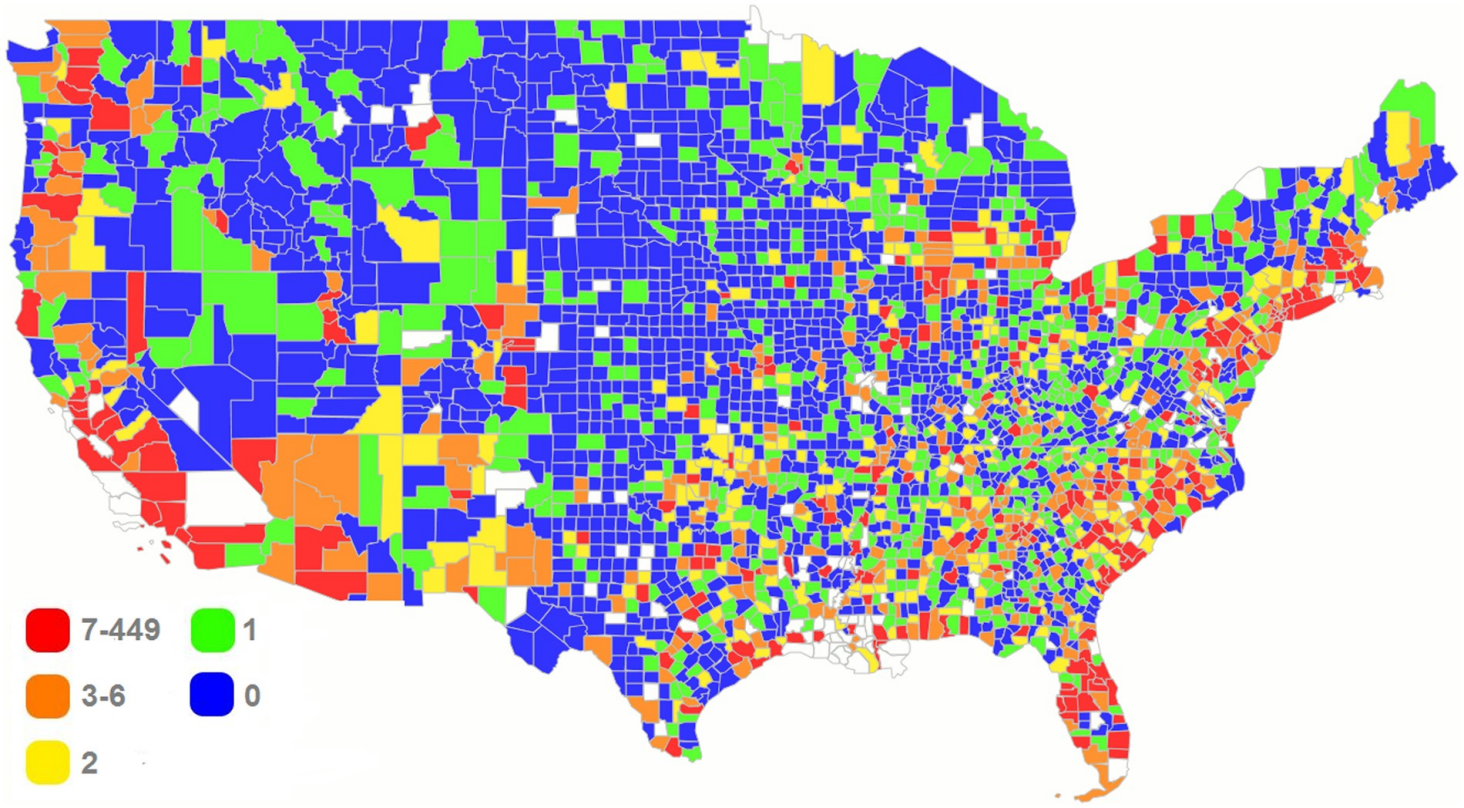
Heat map of total number of gun homicide incidents by county in the contiguous US, 2015. White indicates counties for which no data were available. Created with Mapline mapping software (https://mapline.com), which uses OpenStreetMap geodata (https://openstreetmap.org).

Figs 1 and 2 show county-based heat maps of the gun homicide rates and total numbers of gun homicide incidents in the contiguous US in 2015. A general pattern of higher concentrations of homicide incidents in urban areas is observed. Of all counties, Cook County (in which the city of Chicago is situated) in Illinois had the highest number of incidents (*n* = 449). Patterns are similar between the maps, but more populous areas could have high numbers of incidents yet relatively low homicide rates (e.g., counties in the Miami, Florida, metropolitan area). The final sample spanned 2,729 counties and 70,579 CTs, containing an estimated 314,247,908 individuals, or roughly 98% of the total US population in 2015. The analyses were based on 13,060 firearm-related homicides reported in 2015 in the US, closely approximating (within 1%) the 12,979 firearm-related homicides in 2015 reported by the US Centers for Disease Control and Prevention (CDC) Wonder online database based on data provided by the 57 vital statistics jurisdictions through the Vital Statistics Cooperative Program [2]. In total, 11,385 firearm homicide incidents took place in 8,811 CTs (12.7% of all CTs), with 11,244 non-mass shootings occurring in 8,673 CTs (maximum of 17 incidents in a CT) and 141 mass shootings taking place in 138 CTs (maximum of 2 incidents in a CT). Across counties, the Gini coefficient ranged from 0.16 (Los Alamos County, New Mexico) to 0.81 (Fulton County, Georgia). State and local spending on welfare ranged from $475 per capita (Alabama) to $1,559 per capita (Massachusetts), and state and local spending on education varied from $2,041 per capita (Tennessee) to $4,141 per capita (Wyoming) in 2009 US dollars, based on state population. The racial segregation index ranged from 0 (Norton CZ, Kansas) to 0.55 (Chamberlain CZ, South Dakota), and the county-level social mobility index varied from 23.7 (Richmond City County, Virginia) to 63.8 (Stark County, North Dakota). Additional descriptive statistics are provided for the key social determinants examined in S1 Table. Less than 5% of observations were missing across social determinants and covariates for US populated areas. These missing data were handled using complete case analysis.

Table 1 presents results from modeling social determinants of firearm homicide rates, controlling for all covariates. A 1-SD increase in the county Gini coefficient was associated with an 8% increase in the CT firearm homicide rate (IRR = 1.08, 95% CI 1.01–1.15, *p* = 0.02). Meanwhile, 1-SD increases in welfare spending and education spending were linked to 14% and 22% lower homicide rates, respectively (e.g., for welfare spending: IRR = 0.86, 95% CI 0.83–0.90, *p <* 0.001). Racial segregation and income segregation were related to 2%–3% and 5% higher homicide rates, respectively (Models 1 and 2). A 1-SD increase in institutional social capital was associated with a 19% lower homicide rate (Model 3: IRR = 0.81, 95% CI 0.73–0.91, *p <* 0.001). The Gini coefficient was unchanged in magnitude in this model, which controlled for social capital (Model 3: IRR = 1.08, 95% CI 1.02–1.14, *p* = 0.01). By contrast, the welfare spending effect estimate was markedly attenuated (Model 3: IRR = 1.03, 95% CI 0.92–1.14, *p* = 0.65). An increase in intergenerational social mobility was linked to a 25% lower homicide rate (Model 4: IRR = 0.75, 95% CI 0.66–0.86, *p <* 0.001). Compared to the model that did not control for social mobility, the Gini coefficient effect estimate was marginally attenuated in this model (Model 4: IRR = 1.06, 95% CI 1.01–1.11, *p* = 0.03).

**Table 1:** Social determinants and risk of firearm-related homicide at the CT level in the contiguous US, 2015.

Social determinant	Model 1		Model 2		Model 3		Model 4	
	IRR (95% CI)	*p*-Value	IRR (95% CI)	*p*-Value	IRR (95% CI)	*p*-Value	IRR (95% CI)	*p*-Value
**County level**								
Gini coefficient	1.08 (1.01, 1.15)	0.02	1.07 (1.01, 1.14)	0.02	1.08 (1.02, 1.14)	0.01	1.06 (1.01, 1.11)	0.03
Community social capital	—	—	—	—	0.96 (0.86, 1.08)	0.50	0.99 (0.88, 1.10)	0.83
Institutional social capital	—	—	—	—	0.81 (0.73, 0.91)	<0.001	0.82 (0.74, 0.91)	<0.001
Social mobility	—	—	—	—	—	—	0.75 (0.66, 0.86)	<0.001
**CZ level**								
Racial segregation	1.03 (0.96, 1.12)	0.39	1.02 (0.93, 1.10)	0.73	1.01 (0.92, 1.10)	0.87	1.01 (0.93, 1.10)	0.79
Income segregation	—	—	1.05 (0.95, 1.16)	0.36	1.05 (0.95, 1.17)	0.35	1.02 (0.92, 1.13)	0.67
**State and local level**								
Welfare spending	0.86 (0.83, 0.90)	<0.001	0.86 (0.83, 0.90)	<0.001	1.03 (0.92, 1.14)	0.65	1.00 (0.89, 1.11)	0.97
Education spending	0.78 (0.63, 0.97)	0.03	0.90 (0.61, 1.31)	0.57	1.08 (0.71, 1.65)	0.73	0.74 (0.50, 1.09)	0.13
Protection spending	0.94 (0.83, 1.07)	0.32	0.93 (0.82, 1.06)	0.26	0.97 (0.85, 1.11)	0.66	0.94 (0.84, 1.06)	0.33
**CT level**								
Percent unemployed	1.05 (1.02, 1.08)	<0.001	1.05 (1.02, 1.08)	<0.001	1.05 (1.02, 1.08)	0.002	1.04 (1.01, 1.07)	0.01
Percent on cash assistance	1.11 (1.08, 1.14)	<0.001	1.11 (1.08, 1.14)	<0.001	1.11 (1.08, 1.14)	<0.001	1.10 (1.07, 1.14)	<0.001
Percent in poverty	1.27 (1.15, 1.40)	<0.001	1.27 (1.15, 1.41)	<0.001	1.27 (1.15, 1.40)	<0.001	1.26 (1.14, 1.38)	<0.001
Percent in poverty, squared	0.89 (0.84, 0.94)	<0.001	0.89 (0.83, 0.94)	<0.001	0.89 (0.84, 0.94)	<0.001	0.89 (0.84, 0.94)	<0.001
Percent males living alone	1.12 (1.08, 1.16)	<0.001	1.12 (1.08, 1.16)	<0.001	1.12 (1.09, 1.16)	<0.001	1.12 (1.08, 1.16)	<0.001

IRRs (95% CIs) and *p*-values are derived from multivariate-adjusted negative binomial regression models and correspond to a 1-SD change. All models are adjusted for state fixed effects, total state and local spending, and state gun control policy indicators for concealed weapon carry laws, requirements for gun dealers to report records to the state, and state background check laws. At the CZ level, all models are adjusted for median household income, percentage black, and an indicator variable for whether the CZ corresponded to an urban area. At the county level, all models are adjusted for median household income, percentage black, population density, and property crime rate. At the CT level, all models are adjusted for median household income, (median household income)^2^, percentage with high school education, (percentage with high school education)^2^, percentage black, (percentage black)^2^, percentage male, percentage age 20–34 years, (percentage age 20–34 years)^2^, total population in the year 2012, and (total population in the year 2012)^2^.

CI, confidence interval; CT, census tract; CZ, commuting zone; IRR, incidence rate ratio.

Similar patterns were observed for social determinants in relation to the total numbers of gun homicide incidents (combined non-mass and mass shootings) and non-mass gun homicide incidents separately (Tables 2 and 3, respectively). A 1-SD increase in the county Gini coefficient was associated with a 10% higher total number of firearm-related homicide incidents and non-mass shooting homicide incidents (e.g., Model 1: IRR = 1.10, 95% CI 1.03–1.17, *p* = 0.003; Table 2). Higher welfare spending was linked to a 17%–19% lower incident count (e.g., Model 1, for total combined incidents: IRR = 0.83, 95% CI 0.79–0.87, *p <* 0.001; Table 2). In addition, a 1-SD increase in institutional social capital was related to a 16%–17% lower homicide incident count (e.g., Model 3: IRR = 0.83, 95% CI 0.73–0.95, *p* = 0.01; Table 2). Compared to the model that did not control for social capital (Model 2), the Gini coefficient effect estimate was unchanged in magnitude in this model (Model 3). Meanwhile, the welfare spending estimates were attenuated (e.g., from a 19% to a 4% lower non-mass shooting incident count). An increase in social mobility was associated with a 24% lower incident count (e.g., Model 4, for non-mass shooting incidents: IRR = 0.76, 95% CI 0.67–0.87, *p <* 0.001; Table 3), while the county Gini coefficient estimate was slightly attenuated.

**Table 2:** Social determinants and the total number of firearm-related homicide incidents at the CT level in the contiguous US, 2015.

Social determinant	Model 1		Model 2		Model 3		Model 4	
	IRR (95% CI)	*p*-Value	IRR (95% CI)	*p*-Value	IRR (95% CI)	*p*-Value	IRR (95% CI)	*p*-Value
**County level**								
Gini coefficient	1.10 (1.03, 1.17)	0.003	1.09 (1.03, 1.16)	0.003	1.10 (1.04, 1.16)	<0.001	1.08 (1.03, 1.13)	0.002
Community social capital	—	—	—	—	0.92 (0.83, 1.03)	0.15	0.94 (0.85, 1.05)	0.29
Institutional social capital	—	—	—	—	0.83 (0.73, 0.95)	0.01	0.84 (0.74, 0.95)	0.01
Social mobility	—	—	—	—	—	—	0.76 (0.67, 0.87)	<0.001
**CZ level**								
Racial segregation	1.03 (0.94, 1.12)	0.54	1.01 (0.92, 1.11)	0.90	1.00 (0.90, 1.10)	0.93	1.00 (0.91, 1.10)	0.97
Income segregation	—	—	1.05 (0.94, 1.18)	0.35	1.05 (0.94, 1.18)	0.40	1.02 (0.91, 1.14)	0.70
**State and local level**								
Welfare spending	0.83 (0.79, 0.87)	<0.001	0.83 (0.79, 0.87)	<0.001	0.99 (0.87, 1.12)	0.85	0.95 (0.84, 1.08)	0.47
Education spending	0.97 (0.75, 1.25)	0.80	1.13 (0.75, 1.70)	0.57	1.26 (0.79, 2.01)	0.33	0.86 (0.55, 1.34)	0.51
Protection spending	0.90 (0.79, 1.01)	0.08	0.89 (0.79, 1.00)	0.05	0.92 (0.81, 1.04)	0.20	0.90 (0.80, 1.01)	0.07
**CT level**								
Percent unemployed	1.04 (1.01, 1.07)	0.004	1.04 (1.01, 1.07)	0.003	1.04 (1.01, 1.07)	0.01	1.03 (1.00, 1.06)	0.03
Percent on cash assistance	1.11 (1.08, 1.14)	<0.001	1.11 (1.08, 1.14)	<0.001	1.11 (1.08, 1.14)	<0.001	1.10 (1.07, 1.13)	<0.001
Percent in poverty	1.34 (1.20, 1.48)	<0.001	1.34 (1.20, 1.49)	<0.001	1.34 (1.21, 1.48)	<0.001	1.32 (1.19, 1.46)	<0.001
Percent in poverty, squared	0.83 (0.78, 0.89)	<0.001	0.83 (0.78, 0.89)	<0.001	0.83 (0.78, 0.89)	<0.001	0.84 (0.79, 0.89)	<0.001
Percent males living alone	1.12 (1.08, 1.16)	<0.001	1.12 (1.08, 1.16)	<0.001	1.12 (1.08, 1.16)	<0.001	1.12 (1.08, 1.16)	<0.001

IRRs (95% CIs) and *p*-values are derived from multivariate-adjusted negative binomial regression models and correspond to a 1-SD change. All models are adjusted for state fixed effects, total state and local spending, and state gun control policy indicators for concealed weapon carry laws, requirements for gun dealers to report records to the state, and state background check laws. At the CZ level, all models are adjusted for median household income, percentage black, and an indicator variable for whether the CZ corresponded to an urban area. At the county level, all models are adjusted for median household income, percentage black, population density, and property crime rate. At the CT level, all models are adjusted for median household income, (median household income)^2^, percentage with high school education, (percentage with high school education)^2^, percentage black, (percentage black)^2^, percentage male, percentage age 20–34 years, (percentage age 20–34 years)^2^, total population in the year 2012, and (total population in the year 2012)^2^.

CI, confidence interval; CT, census tract; CZ, commuting zone; IRR, incidence rate ratio.

**Table 3:** Social determinants and the total number of non-mass shooting homicide incidents at the CT level in the contiguous US, 2015.

Social determinant	Model 1		Model 2		Model 3		Model 4	
	IRR (95% CI)	*p*-Value	IRR (95% CI)	*p*-Value	IRR (95% CI)	*p*-Value	IRR (95% CI)	*p*-Value
**County level**								
Gini coefficient	1.10 (1.03, 1.17)	0.003	1.09 (1.03, 1.16)	0.002	1.10 (1.05, 1.16)	<0.001	1.08 (1.03, 1.13)	0.001
Community social capital	—	—	—	—	0.92 (0.82, 1.02)	0.12	0.94 (0.84, 1.04)	0.24
Institutional social capital	—	—	—	—	0.84 (0.73, 0.96)	0.01	0.85 (0.74, 0.96)	0.01
Social mobility	—	—	—	—	—	—	0.76 (0.67, 0.87)	<0.001
**CZ level**								
Racial segregation	1.03 (0.94, 1.12)	0.55	1.01 (0.91, 1.11)	0.88	1.00 (0.90, 1.10)	0.96	1.00 (0.91, 1.10)	0.94
Income segregation	—	—	1.05 (0.94, 1.17)	0.39	1.05 (0.93, 1.17)	0.44	1.02 (0.91, 1.14)	0.76
**State and local level**								
Welfare spending	0.81 (0.77, 0.85)	<0.001	0.81 (0.77, 0.85)	<0.001	0.96 (0.84, 1.10)	0.56	0.93 (0.82, 1.06)	0.27
Education spending	1.06 (0.82, 1.38)	0.64	1.23 (0.81, 1.86)	0.34	1.35 (0.84, 2.18)	0.22	0.92 (0.58, 1.45)	0.72
Protection spending	0.89 (0.79, 1.01)	0.07	0.88 (0.78, 1.00)	0.04	0.92 (0.81, 1.04)	0.17	0.89 (0.79, 1.00)	0.06
**CT level**								
Percent unemployed	1.04 (1.01, 1.07)	0.005	1.04 (1.01, 1.07)	0.005	1.04 (1.01, 1.07)	0.01	1.03 (1.00, 1.06)	0.03
Percent on cash assistance	1.11 (1.08, 1.14)	<0.001	1.11 (1.08, 1.14)	<0.001	1.11 (1.08, 1.14)	<0.001	1.10 (1.07, 1.13)	<0.001
Percent in poverty	1.34 (1.21, 1.49)	<0.001	1.35 (1.21, 1.49)	<0.001	1.34 (1.21, 1.49)	<0.001	1.33 (1.20, 1.47)	<0.001
Percent in poverty, squared	0.83 (0.78, 0.89)	<0.001	0.83 (0.78, 0.88)	<0.001	0.83 (0.78, 0.88)	<0.001	0.83 (0.78, 0.89)	<0.001
Percent males living alone	1.12 (1.08, 1.16)	<0.001	1.12 (1.08, 1.16)	<0.001	1.12 (1.09, 1.16)	<0.001	1.12 (1.08, 1.17)	<0.001

IRRs (95% CIs) and *p*-values are derived from multivariate-adjusted negative binomial regression models and correspond to a 1-SD change. All models are adjusted for state fixed effects, total state and local spending, and state gun control policy indicators for concealed weapon carry laws, requirements for gun dealers to report records to the state, and state background check laws. At the CZ level, all models are adjusted for median household income, percentage black, and an indicator variable for whether the CZ corresponded to an urban area. At the county level, all models are adjusted for median household income, percentage black, population density, and property crime rate. At the CT level, all models are adjusted for median household income, (median household income)^2^, percentage with high school education, (percentage with high school education)^2^, percentage black, (percentage black)^2^, percentage male, percentage age 20–34 years, (percentage age 20–34 years)^2^, total population in the year 2012, and (total population in the year 2012)^2^.

CI, confidence interval; CT, census tract; CZ, commuting zone; IRR, incidence rate ratio.

The analysis of mass homicide incidents alone (Table 4) revealed effect estimates that were accompanied by much wider confidence intervals than those for the previous respective findings. Although 1-SD increases in income inequality and in social mobility were linked to a 15% higher and a 26% lower total number of firearm-related mass homicide incidents in a CT, respectively, the *p*-values for both of these associations exceeded 0.10. An increase in welfare spending was also unrelated to a change in the incident count. By contrast, an increase in institutional social capital was associated with a 29% lower homicide incident count (Model 3: IRR = 0.71, 95% CI 0.52–0.98, *p* = 0.04).

**Table 4:** Social determinants and the total number of mass shooting homicide incidents at the CT level in the contiguous US, 2015.

Social determinant	Model 1		Model 2		Model 3		Model 4	
	IRR (95% CI)	*p*-Value	IRR (95% CI)	*p*-Value	IRR (95% CI)	*p*-Value	IRR (95% CI)	*p*-Value
**County level**								
Gini coefficient	1.15 (0.94, 1.40)	0.18	1.13 (0.93, 1.37)	0.22	1.12 (0.93, 1.35)	0.24	1.09 (0.90, 1.32)	0.38
Community social capital	—	—	—	—	1.11 (0.74, 1.67)	0.63	1.17 (0.76, 1.81)	0.47
Institutional social capital	—	—	—	—	0.71 (0.52, 0.98)	0.04	0.74 (0.53, 1.02)	0.07
Social mobility	—	—	—	—	—	—	0.74 (0.49, 1.12)	0.15
**CZ level**								
Racial segregation	0.86 (0.64, 1.16)	0.32	0.80 (0.55, 1.15)	0.22	0.78 (0.54, 1.14)	0.19	0.78 (0.54, 1.11)	0.16
Income segregation	—	—	1.22 (0.86, 1.73)	0.26	1.22 (0.86, 1.73)	0.27	1.20 (0.85, 1.69)	0.30
**State and local level**								
Welfare spending	1.03 (0.75, 1.42)	0.85	1.05 (0.76, 1.44)	0.77	1.07 (0.76, 1.49)	0.71	1.02 (0.71, 1.46)	0.92
Education spending	0.76 (0.44, 1.29)	0.31	0.73 (0.41, 1.28)	0.27	0.74 (0.42, 1.31)	0.30	0.72 (0.41, 1.28)	0.27
Protection spending	0.86 (0.58, 1.29)	0.47	0.84 (0.56,1.26)	0.40	0.81 (0.52, 1.27)	0.36	0.81 (0.52, 1.26)	0.36
**CT level**								
Percent unemployed	1.15 (0.95, 1.41)	0.16	1.16 (0.95, 1.41)	0.15	1.15 (0.95, 1.41)	0.16	1.14 (0.94, 1.39)	0.19
Percent on cash assistance	0.98 (0.73, 1.32)	0.90	0.97 (0.72, 1.32)	0.87	0.98 (0.72, 1.33)	0.89	0.97 (0.71, 1.32)	0.85
Percent in poverty	1.09 (0.60, 1.99)	0.77	1.10 (0.61, 2.00)	0.74	1.10 (0.61, 1.96)	0.76	1.08 (0.60, 1.95)	0.79
Percent in poverty, squared	0.89 (0.56, 1.42)	0.64	0.88 (0.56, 1.40)	0.60	0.89 (0.57, 1.39)	0.61	0.89 (0.57, 1.40)	0.62
Percent males living alone	0.91 (0.72, 1.15)	0.44	0.91 (0.72, 1.15)	0.43	0.91 (0.73, 1.15)	0.44	0.91 (0.72, 1.15)	0.43

IRRs (95% CIs) and *p*-values are derived from multivariate-adjusted negative binomial regression models and correspond to a 1-SD change. All models are adjusted for census statistical division fixed effects, total state and local spending, and state gun control policy indicators for concealed weapon carry laws, requirements for gun dealers to report records to the state, and state background check laws. At the CZ level, all models are adjusted for median household income, percentage black, and an indicator variable for whether the CZ corresponded to an urban area. At the county level, all models are adjusted for median household income, percentage black, population density, and property crime rate. At the CT level, all models are adjusted for median household income, (median household income)^2^, percentage with high school education, (percentage with high school education)^2^, percentage black, (percentage black)^2^, percentage male, percentage age 20–34 years, (percentage age 20–34 years)^2^, total population in the year 2012, and (total population in the year 2012)^2^.

CI, confidence interval; CT, census tract; CZ, commuting zone; IRR, incidence rate ratio.

At the CT level, 1-SD increases in the percentage of residents in poverty, percentage who were unemployed, percentage who were receiving cash assistance, and percentage of male residents who lived alone were all linked to higher gun homicide rates and higher numbers of incidents of non-mass shooting homicides (Tables 1–3). A 1-SD increase in the percentage of residents in poverty was associated with a 26%–27% higher rate of homicides, and a 1-SD increase in the percentage of male residents who lived alone was related to a 12% higher homicide rate (Table 1).

In generalized linear regression models that explored potential mediation (i.e., whether the exposure was correlated with the respective potential mediator, to evaluate the remaining step of the Baron and Kenny approach), the county Gini coefficient was inversely associated with levels of social mobility and welfare spending, and welfare spending had a positive relationship with institutional social capital (see S6 Table). For the causal mediation analysis approach [51], slightly more parsimonious models (i.e., omitting all quadratic terms, and using fixed effects for census statistical divisions rather than states) were required for models to converge. The estimated percentages of the total effects for income inequality mediated by welfare spending, institutional social capital, and social mobility ranged from 3.2% to 32.3%, while the estimated percentage of the total effect for welfare spending mediated by institutional social capital was 21.4% (see S7–S10 Tables). All 95% confidence intervals for the percentages due to mediation included 0. The estimated 32.3% of the total effect for income inequality mediated by social mobility had the smallest associated *p*-value, 0.055, although the confidence interval was wide (95% CI −0.64%, 65.26%; S9 Table). All estimated exposure–mediator interactions were non-significant at the 5% level, lending support to the validity of the Baron and Kenny approach.

In sensitivity analyses, the above findings were robust after excluding 2 CTs with outlying values for homicide rates. Similar results were also obtained when the definition of a non-mass shooting was modified to an incident in which the shooter killed up to 3 rather than 2 persons (see S11 Table). In addition, the findings were robust (with identical IRRs to 2 decimal places) when protection spending was limited to police protection services only, and when supplementary neighborhood demographic and socioeconomic factors were added individually to the models. In analyses based on the state-level Gini coefficient with a 15-year lag period, the magnitudes of the estimates were similar, but all *p*-values were larger compared to estimates obtained for the county-level Gini coefficient using a 17-year lag (S3 Table).

Finally, S12–S15 Tables present the main results in Tables 1–4 based on rescaling of social determinant measures using alternative meaningful units instead of a 1-SD change.

## Discussion

### Principal findings

Spanning 95% of CTs in the contiguous US, this study found that several social determinants of health—county-level institutional social capital and social mobility, state and local welfare spending, and income inequality—were associated with CT-level gun homicide rates along with the numbers of combined non-mass and mass homicide incidents and non-mass homicide incidents alone. Welfare spending, institutional social capital, and social mobility exhibited the most robust associations, while upward social mobility showed the largest effects—with a 1-SD increase related to a 25% reduction in the homicide rate and a 24% reduction in the number of non-mass shootings. There was also evidence to support that social mobility mediated nearly one-third of the effects of income inequality and that social capital mediated roughly one-fifth of the effects of welfare spending, although these estimates were imprecise. At the CT level, the percentages of residents in poverty and of male residents who lived alone were further linked to 26%–27% and 12% higher gun homicide rates, respectively. By contrast, of the social determinants examined, only institutional social capital was associated with the total number of mass shootings. Meanwhile, there was limited evidence of relationships for other social determinants including racial and income segregation.

### What this study adds and its findings in relation to other studies

A variety of social determinants of health have been theoretically and empirically linked to health outcomes. Social capital has demonstrated relationships with physical and mental health, suggesting that it exerts health-promoting effects at the community level over and above the effects of individual-level factors [54,55]. One posited explanation draws on disorganization theory, whereby trust between neighbors enables communities to act collectively against local problems such as disorderliness and criminal behaviors [43,56]. In disadvantaged communities, a lack of trust in institutions, such as police, to treat residents with respect may further lead residents to believe that the formal apparatus of social control is unjust, resulting in a greater willingness to take the law into their own hands [57–59]. Meanwhile, income inequality has been shown to be associated with poor health outcomes including mortality [60]. Through greater perceptions of relative deprivation, income inequality may induce feelings of resentment, and cause individuals to resort to criminal behaviors including homicide [61]. Residential racial segregation and economic segregation have been linked to infant mortality and adverse birth outcomes [62,63]. Both forms of segregation may adversely impact health by spatially concentrating the poor, leading to reduced educational and employment opportunities and less access to local health-related resources such as options for purchasing healthy foods [62]. Non-medical social spending has also been found to be related to mortality, and is conceivably linked to population health. For example, government spending on public goods such as education and social assistance can improve socioeconomic conditions, and thereby may serve as investment in the social determinants of health [21]. Moreover, the added economic security that welfare benefits provide could plausibly stave off disadvantage, a risk factor for committing crimes. Finally, lower intergenerational social mobility has been linked to worse health behaviors and mortality [12,64]. Reduced economic opportunity may diminish economic and social returns to health investments or spur individuals to lose a sense of hope, which in turn could undermine health and health behaviors [12,65,66]. All of these social determinants of health are further believed to be mechanistically intertwined. For example, income inequality is posited to be a driver of social mobility [11], social capital [60,67], and welfare spending [67], and social capital may mediate the relationship between social spending and health [34]. In addition, racial and income segregation could hinder upward mobility through spatial mismatches in employment, such as by making it harder to physically access jobs or resources that facilitate social mobility [11].

These social determinants of health have been investigated and linked to homicide rates to varying degrees. Ecological, cross-sectional data suggest inverse relationships between social capital and overall homicide rates and firearm homicide rates in some studies [68–70] yet not in other studies [71,72]. Ecological evidence also points towards distrust in public institutions and erosion of trust among citizens as being possible drivers of mass shootings and homicide rates [73]. Likewise, to date, findings from empirical studies of income inequality and homicide have been mixed. Some studies have confirmed the theorized positive association [22,74], whereas other studies have found no association [75] or even an inverse relationship [61,76,77]. Possible explanations for this mixed evidence include the ecological and cross-sectional design of many of the studies, a study design which is prone to confounding and reverse causation. Several US-based ecological studies have also investigated racial segregation in relation to homicides, primarily in black individuals but also in Hispanic and white individuals [62,78–84]. Most of these studies have used the dissimilarity index, a racial segregation measure representing the percentage of a group’s population that would have to move for each neighborhood to have the same percentage of that group as the entire metropolitan area. In a state-level ecological study, a higher level of dissimilarity was linked to a higher homicide rate among black individuals yet a lower homicide rate among white individuals [81]. Other studies have found positive or null associations between racial segregation and homicide rates in Hispanic individuals [82–84]. Importantly, no racial segregation studies to date appear to have focused on firearm-related homicides. In primarily ecological studies, inverse relationships between social spending and violent crime, homicides, and external injuries have been observed [21,30,85]. Finally, only 1 study has identified county-level social mobility to be correlated with violent crime rates [11].

The findings from the present study are generally in line with previous work on the association between income inequality and violent crimes including firearm-related homicides in the US. A previous study found a strong correlation of 0.76 between state-level income inequality and the violent crime rate involving firearms [69]. In a study among Chicago neighborhoods, there was a similar correlation of 0.75 between income inequality and the homicide rate [86]. A recent ecological, lagged cross-sectional study [22] observed a positive association between US county-level income inequality and the homicide rate, although the association only attained significance at the 5% level in black individuals. Each 1-SD unit increase in the county-level Gini coefficient was related to a 9% increase in the county-level firearm homicide rate among black individuals 5 to 15 years later, controlling for county-level age, gender, and race/ethnicity composition, crime rate, deprivation, social capital, urbanicity, and firearm ownership [22]. Finally, a multilevel analysis [21] determined that a higher state-level Gini coefficient was associated with a lower individual probability of external injuries including homicide and excluding suicide. With the exception of this last study, all past studies have been ecological in design at the state, county, or neighborhood level. Moreover, while social capital has been proposed as a mediating factor for the effects of income inequality on health [60,67], the current study found no evidence to support such a pathway.

This study’s findings of relationships between social mobility and gun homicides are also in keeping with recent evidence of deleterious associations between low county-level social mobility and all-cause mortality and health behaviors [12,64] as well as ecological evidence of associations between institutional social capital and homicide rates [58,87]. By contrast, the present study found no associations for community social capital. Previous ecological cross-sectional studies using similar formal/civic participation indicators have yielded mixed evidence [68–72]. In 2 ecological studies—a state-level study and a cross-national study among 31 OECD nations—higher total government spending was linked to lower homicide rates [30,85]. For example, each $10,000 spent by a US state, per person in poverty, on social services (including education and welfare benefits), transportation, the environment, public safety, and housing was associated with a 16.4% decrease in the average homicide rate at the state level 1 year later (*p <* 0.001) [30]. Finally, a multilevel analysis that adopted an instrumental variable analysis approach to strengthen causal inference [21] determined that higher public welfare and education spending was related to a lower probability of external injuries including homicide. Unlike previous ecological studies, the present study accounted for social determinants at multiple levels and hence reduced the likelihood of bias due to residual confounding.

### Strengths and limitations of the study

This study has several strengths, including its simultaneous examination of multiple social determinants, which to the author’s knowledge enabled the first direct comparisons of effects along the same scales, with adjustment for the same controls; use of plausible lag periods for effects, which permitted a temporal assumption in support of causation; examination of data at multiple geographic levels, thereby enabling control for CT compositional factors as well as confounding factors at other geographic levels to avoid ecological fallacy; inclusion of data for the vast majority of CTs in the contiguous US, thereby enhancing generalizability; and analysis of non-mass shootings and mass shootings separately to identify whether there were common social determinant drivers. Moreover, the analyses explored the potential mediation of effects, and assessed goodness-of-fit statistics and alternative specifications for variables to minimize model mis-specification bias.

Nonetheless, there are limitations to this study. First, while multiple factors at multiple levels were incorporated into the analyses, residual confounding is still possible, including by factors at the individual/household level as well as area-level factors such as the location of residence. Second, although the analyses accounted for lagged effects, they did not account for time-varying effects and confounders, such as those related to selective migration into or out of CTs over time, changes in residential segregation/gentrification over time, or rapid economic shifts during the Great Recession, which occurred between December 2007 and June 2009. Although the study omitted time-varying covariates, the population size on average several years prior to the year of examined homicides (2015) was taken into account by controlling for the CT population size centered in 2012. Third, while results from a range of lag periods were obtained for social spending (from 5 to 10 years) and income inequality (from 3 to 17 years), the exploration of multiple lag periods for other social determinants was limited by the availability of data. More salient specific lag periods (such as for racial and income segregation) may hence have been missed.

Fourth, while this study advances the scope of concurrent empirical examination of social determinants, the exclusion of complex, multilevel, and reciprocal interactions cannot be ruled out. Reciprocal interactions can include feedback loops that characterize complex systems. For example, income inequality may lead to reduced welfare spending, and, in turn, reduced income supports to low-income households due to low welfare spending could feed back dynamically and exacerbate levels of income inequality. Systems science/complex systems approaches such as agent-based modeling and system dynamics are designed to incorporate such complex nonlinearities and feedback loops into the modeling process [88]. In addition, cross-level interactions may exist between specific macro-level social determinants and characteristics of neighborhoods in their associations with homicides. For example, if low county-level social mobility magnifies the impacts of social and economic conditions such as job prospects for residents in poor neighborhoods (i.e., county-level social mobility interacts with neighborhood socioeconomic deprivation), we might expect to see greater homicide rates in such neighborhoods than expected based on these factors’ independent contributions.

Fifth, the geocoded dataset from the GVA does not allow for disaggregation by important factors such as age, race/ethnicity, gender, and whether homicides were perpetrated by law enforcement versus other individuals; for each of these characteristics, the roles of social determinants might vary. Nevertheless, roughly 7.5% of all gun homicides in 2015 in the 48 contiguous US states were reportedly by police in 2015 [89]. The magnitude of this proportion along with the relatively large associations of gun homicides with welfare spending, institutional social capital, and social mobility suggest that law enforcement-related homicides were not the principal drivers of the observed associations. Researchers have further recommended that future homicide studies conduct analyses for racial groups separately, since the associations for combined-race homicide rates could mask underlying associations by race [90]. The inability to disaggregate by race in the present study might offer a partial explanation for the null associations found for racial segregation.

Last, with only 141 homicide incidents defined as mass shootings (amounting to 1.2% of the total number of incidents), a lack of statistical power likely constrained the ability to detect associations when mass shootings were examined separately. For social mobility, the effect estimates for mass shootings and non-mass shootings were similar (26% and 24% lower number of incidents, respectively). In addition, over two-thirds (70%) of the incidents classified as mass shootings involved exactly 3 deaths. Given the robust findings for non-mass shootings that included such incidents, and given no plausible reason why social drivers of incidents involving 3 deaths would differ from those involving 4 deaths, some of these null findings are likely to be on a statistical basis. In future research, pooling mass shooting data across multiple years would aid in ruling out inadequate statistical power as a potential explanation and in better elucidating the social drivers of mass shootings.

### Implications of the study and next steps

Rising gaps between the rich and the poor may lead to fewer economic opportunities for upward income mobility [11]. This premise is supported by the study’s findings suggesting mediation of the effects of income inequality by social mobility. In addition, the lack of an apparent impact of more horizontal forms of community social capital implies the greater importance of more vertical forms of social capital, reflecting individuals’ relationships with institutions. According to Gallup polls, Americans’ trust in institutions (a form of institutional social capital), from newspapers to the Supreme Court to Congress, has reached historic lows over the past decade [91], and this trend parallels the rising rates of gun violence. Finally, public assistance programs and state supplements for unemployment insurance, workers’ compensation, and work incentive programs may provide critical social safety nets to ensure socioeconomic security for individuals at risk. In keeping with this study’s findings, public welfare spending may also be a driver of institutional social capital, with its impacts on firearm-related homicides plausibly mediated by social capital.

Overall, with non-mass shootings comprising nearly 99% of all firearm-related homicide incidents and contributing to over 95% of all gun deaths in America in 2015, the findings of this study should serve as a wake-up call for greater investments by researchers and policymakers to better understand and ameliorate levels of income inequality and increase institutional social capital and social mobility to reduce the burden of firearm-related homicides. Candidate policy levers might include economic policies that raise taxation on the very wealthy [92], policies that ensure fair voting and redistricting processes to encourage higher voter turnout, and policies that increase the affordability of college attendance for low-income students [93]. Taxation policies that include redistribution to low-income households would also reduce the percentage of persons in poverty, a neighborhood characteristic found in this study to be a risk factor for both gun homicide rates and higher counts of homicide incidents. If effective, such policies could have marked impacts on reducing homicides.

This study’s findings and its limitations suggest several principal opportunities for future research. First, further studies are needed to elucidate if the observed relationships are truly causal, such as with the application of longitudinal data and quasi-experimental study designs that exploit random variation arising from policies that alter levels of income inequality, welfare spending, institutional social capital, and income mobility. Second, the availability of data at a micro (i.e., individual/household) level would help to delineate underlying behavioral and psychosocial pathways, particularly for institutional social capital and social mobility [12], and enable control for additional potential confounders. Third, future public releases of geolocated gun homicide data should include information for disaggregating homicides by victim and perpetrator characteristics such as race/ethnicity and gender, and whether the homicide was committed by law enforcement. Fourth, recent unfavorable changes in income inequality and social mobility, as well as stalls or falls in life expectancy, have occurred in several Western European nations including the UK [35,94–96] in tandem with the US. In these other developed economies, it would be useful to explore whether social determinants show analogous consequences with respect to violent behaviors, despite firearm-related homicide rates being markedly lower in other developed nations than in the US. For example, both London and other parts of the UK are experiencing a substantial rise in knife crimes [97]. Such comparisons may also confirm the findings from this study suggesting that non-mass shootings and mass shootings are related phenomena that exist along a single continuum with at least one common driver—institutional social capital.

### Conclusions

This study finds that the rich–poor gap, citizens’ levels of trust in institutions, economic opportunity, and public welfare spending are associated with rates of firearm-related homicides in the US. Further elucidating and modifying the social and economic drivers of firearm-related homicides and mass shootings—possibly through candidate levers including taxation policies and policies that increase college affordability—are essential if the US as a nation is to effectively address the epidemic of gun homicides, and reverse recent trends in life expectancy among Americans.

## Supporting information

S1 STROBE Statement (PDF)

S1 Table Descriptive characteristics of key social determinants examined and the total number of firearm-related homicide incidents at the CT level, 2015. (PDF)

S2 Table Social determinants, data sources, average lag periods in prior studies, and temporal order for examining mediation in present study. (PDF)

S3 Table Results from analysis of varying lag periods for county-level income inequality and state-level income inequality (per 0.10-unit increase in the Gini coefficient) and risk of firearm-related homicide and total number of firearm-related homicide incidents at the CT level in the contiguous US, 2015. (PDF)

S4 Table Results from analysis of varying lag periods for state and local welfare and education spending (per $250 per capita increase) and risk of firearm-related homicide and total number of firearm-related homicide incidents at the CT level in the contiguous US, 2015. (PDF)

S5 Table Variance inflation factors for social determinant exposure variables. (PDF)

S6 Table Coefficient estimates from generalized linear regression models exploring potential mediation pathways. (PDF)

S7 Table Causal mediation analysis of institutional social capital as a mediator of the association between income inequality and firearm-related homicide rate. (PDF)

S8 Table Causal mediation analysis of welfare spending as a mediator of the association between income inequality and firearm-related homicide rate. (PDF)

S9 Table Causal mediation analysis of social mobility as a mediator of the association between income inequality and firearm-related homicide rate. (PDF)

S10 Table Causal mediation analysis of institutional social capital as a mediator of the association between welfare spending and firearm-related homicide rate. (PDF)

S11 Table Social determinants and the total number of non-mass firearm-related homicide incidents at the CT level in the contiguous US, 2015 (using the Federal Bureau of Investigation definition of non-mass versus mass shootings). (PDF)

S12 Table Social determinants and risk of firearm-related homicide at the CT level in the contiguous US, 2015. (PDF)

S13 Table Social determinants and the total number of firearm-related homicide incidents at the CT level in the contiguous US, 2015. (PDF)

S14 Table Social determinants and the total number of firearm-related non-mass homicide incidents at the CT level in the contiguous US, 2015. (PDF)

S15 Table Social determinants and the total number of firearm-related mass homicide incidents at the CT level in the contiguous US, 2015. (PDF)

## Abbreviations

ACS: American Community Survey
CI: confidence interval
CPS: Current Population Survey
CT: census tract
CZ: commuting zone
GVA: Gun Violence Archive
IRR: incidence rate ratio
SD: standard deviation

## References

[1] Beauchamp Z. Guns killed more Americans in 12 years than AIDS, war, and illegal drug overdoses combined. Vox. 2016 Jun 12 [cited 2019 Jun 1]. https://www.vox.com/2015/10/3/9446193/gun-deaths-aids-war-terrorism.

[2] CDC WONDER. Underlying cause of death, 1999–2017 request. Atlanta: Centers for Disease Control and Prevention; 2019 [cited 2019 Nov 13]. https://wonder.cdc.gov/controller/datarequest/D76.

[3] Fowler KA, Dahlberg LL, Haileyesus T, Annest JL. Firearm injuries in the United States. Prev Med. 2015;(79):5–14. 10.1016/j.ypmed.2015.06.002 PMC4700838 26116133

[4] Kalesan B, Mobily ME, Keiser O, Fagan JA, Galea S. Firearm legislation and firearm mortality in the USA: a cross-sectional, state-level study. Lancet. 2016;387(10030):1847–55. 10.1016/S0140-6736(15)01026-0 26972843

[5] Siegel M, Xuan Z, Ross CS, Galea S, Kalesan B, Fleegler E, et al Easiness of legal access to concealed firearm permits and homicide rates in the United States. Am J Public Health. 2017;107:1923–9. 10.2105/AJPH.2017.304057 29048964 PMC5678379

[6] Santaella-Tenorio J, Cerdá M, Villaveces A, Galea S. What do we know about the association between firearm legislation and firearm-related injuries?. Epidemiol Rev. 2016;38(1):140–57. 10.1093/epirev/mxv012 26905895 PMC6283012

[7] Rivara FP, Studdert DM, Wintemute GJ. Firearm-related mortality: a global public health problem. JAMA. 2018;320(8):764–5. 10.1001/jama.2018.9942 30167677

[8] Davis AB, Gaudino JA, Soskolne CL, Al-Delaimy WK, International Network for Epidemiology in Policy. The role of epidemiology in firearm violence prevention: a Policy Brief. Int J Epidemiol. 2018;47(4):1015–9. 10.1093/ije/dyy059 29718257

[9] Hemenway D. Reducing firearm violence. Crime Justice. 2017;46:201–30.

[10] Marmot M, Friel S, Bell R, Houweling TA, Taylor S, Commission on Social Determinants of Health. Closing the gap in a generation: health equity through action on the social determinants of health. Lancet. 2008;372(9650):1661–9. 10.1016/S0140-6736(08)61690-6 18994664

[11] Chetty R, Hendren N, Kline P, Saez E. Where is the land of opportunity? The geography of intergenerational mobility in the United States. Q J Econ. 2014;129:1553–623.

[12] Venkataramani AS, Brigell R, O’Brien R, Chatterjee P, Kawachi I, Tsai AC. Economic opportunity, health behaviors, and health outcomes in the USA: a population-based cross-sectional study. Lancet Public Health. 2016;1(1):e18–25. 10.1016/S2468-2667(16)30005-6 29253376 PMC5947845

[13] Garcia RM, Taylor RB, Lawton BA. Impacts of violent crime and neighborhood structure on trusting your neighbors. Justice Q. 2007;24(4):679–704.

[14] Gun Violence Archive. Mass shootings—2015. Washington (DC): Gun Violence Archive; 2019 [cited 2019 Jun 1]. https://www.gunviolencearchive.org/reports/mass-shootings/2015.

[15] Aufrichtig A. Mapping US gun murders at a micro level: new data zooms in on violence. The Guardian. 2017 Mar 20 [cited 2019 Jun 1]. https://www.theguardian.com/world/2017/mar/20/mapping-gun-murders-micro-level-new-data-2015?CMP.

[16] GovTrack. H.R. 2076 (112th): Investigative Assistance for Violent Crimes Act of 2012. GovTrack; 2013 Jan 2 [cited 2019 Jun 1]. https://www.govtrack.us/congress/bills/112/hr2076/text

[17] Kim D, Griffin BA, Kabeto M, Escarce J, Langa KM, Shih RA. Lagged associations of metropolitan statistical area-and state-level income inequality with cognitive function: the Health and Retirement Study. PLoS ONE. 2016;11(6):e0157327 10.1371/journal.pone.0157327 27332986 PMC4917220

[18] Blakely TA, Kennedy BP, Glass R, Kawachi I. What is the lag time between income inequality and health status? J Epidemiol Community Health. 2000;54(4):318–9. 10.1136/jech.54.4.318 10827916 PMC1731662

[19] United States Census Bureau. American Community Survey. Suitland (MD): United States Census Bureau; 2019 [cited 2019 Nov 13]. https://factfinder.census.gov/faces/nav/jsf/pages/programs.xhtml?program=acs.

[20] Internal Revenue Service. Statistics of income: individual income tax returns, 2012. Technical report. Washington (DC): Internal Revenue Service; 2013.

[21] Kim D. The associations between US state and local social spending, income inequality, and individual all-cause and cause-specific mortality: the National Longitudinal Mortality Study. Prev Med. 2016;84:62–8. 10.1016/j.ypmed.2015.11.013 26607868 PMC5766344

[22] Rowhani-Rahbar A, Quistberg DA, Morgan ER, Hajat A, Rivara FP. Income inequality and firearm homicide in the US: a county-level cohort study. Inj Prev. 2019;25(Suppl 1):i25–30. 10.1136/injuryprev-2018-043080 30782593

[23] Mellor JM, Milyo J. Is exposure to income inequality a public health concern? Lagged effects of income inequality on individual and population health. Health Serv Res. 2003;38:137–51. 10.1111/1475-6773.00109 12650385 PMC1360878

[24] Vice Chairman’s Staff of the Joint Economic Committee. The geography of social capital in America. St. George (UT): Office of Senator Michael S. Lee; 2018 Apr 11 [cited 2019 Jun 1]. https://www.lee.senate.gov/public/index.cfm?a=files.serve&File_id=DA64FDB7-3B2E-40D4-B9E3-07001B81EC31.

[25] United States Census Bureau. Current Population Survey (CPS): supplemental surveys. Suitland (MD): United States Census Bureau; 2018 Apr 30 [cited 2019 Jun 1]. http://www.census.gov/programs-surveys/cps/about/supplemental-surveys.html.

[26] National Center for Charitable Statistics. Guide to using NCCS data. National Center for Charitable Statistics; 2018 Dec 13 [cited 2019 Jun 1]. http://nccs-data.urban.org/NCCS-data-guide.pdf.

[27] US Election Assistance Commission. 2012 Election administration and voting survey: a summary of key findings, September 2013. Silver Spring (MD): US Election Assistance Commission; 2013 Sept [cited 2019 Jun 1]. https://www.eac.gov/assets/1/6/2012ElectionAdministrationandVoterSurvey.pdf.

[28] US Election Assistance Commission. The election administration and voting survey: 2016 comprehensive report. Silver Spring (MD): US Election Assistance Commission; 2016 [cited 2019 Jun 1]. https://www.eac.gov/assets/1/6/2016_EAVS_Comprehensive_Report.pdf.

[29] United States Census Bureau. Annual survey of state & local government finances. Suitland (MD): United States Census Bureau; 2019 [cited 2019 Jun 1]. http://www.census.gov/econ/overview/go0400.html.

[30] Sipsma HL, Canavan ME, Rogan E, Taylor LA, Talbert-Slagle KM, Bradley EH. Spending on social and public health services and its association with homicide in the USA: an ecological study. BMJ Open. 2017;7(10):e016379 10.1136/bmjopen-2017-016379 29025831 PMC5652551

[31] Phillips JA. Explaining discrepant findings in cross-sectional and longitudinal analyses: an application to US homicide rates. Soc Sci Res. 2006;35(4):948–74.

[32] Graif C, Sampson RJ. Spatial heterogeneity in the effects of immigration and diversity on neighborhood homicide rates. Homicide Stud. 2009;13(3):242–60. 10.1177/1088767909336728 20671811 PMC2911240

[33] Votruba ME, Kling JR. Effects of neighborhood characteristics on the mortality of black male youth: evidence from Gautreaux, Chicago. Soc Sci Med. 2009;68(5):814–23. 10.1016/j.socscimed.2008.12.018 19155115

[34] Jenson J, Saint-Martin D. New routes to social cohesion? Citizenship and the social investment state. Can J Sociol. 2003;1:77–99.

[35] Krueger AB. The rise and consequences of inequality in the United States. Washington (DC): Obama White House Archives; 2012 Jan 12 [cited 2019 Jun 1]. https://obamawhitehouse.archives.gov/sites/default/files/krueger_cap_speech_final_remarks.pdf.

[36] Kumlin S, Rothstein B. Making and breaking social capital: the impact of welfare state institutions. Comp Polit Stud. 2005;38(4):339–65.

[37] Kääriäinen J, Lehtonen H. The variety of social capital in welfare state regimes—a comparative study of 21 countries. European Societies. 2006;8(1):27–57.

[38] Iceland J. Beyond black and white: metropolitan residential segregation in multiethnic America. Soc Sci Res. 2004;33(2):248–71.

[39] Reardon SF, Firebaugh G. Measures of multigroup segregation. Sociol Methodol. 2002;32(1):33–67.

[40] Fleegler EW, Lee LK, Monuteaux MC, Hemenway D, Mannix R. Firearm legislation and firearm-related fatalities in the United States. JAMA Intern Med. 2013;173(9):732–40. 10.1001/jamainternmed.2013.1286 23467753

[41] Armstead TL, Wilkins N, Nation M. Structural and social determinants of inequities in violence risk: a review of indicators. J Community Psychol. 2019 8 17 10.1002/jcop.22232 31421656 PMC7278040

[42] Branas CC, Richmond TS, Schwab CW. Firearm homicide and firearm suicide: opposite but equal. Public Health Rep. 2004;119(2):114–24. 10.1177/003335490411900203 15192897 PMC1497617

[43] Pratt TC, Cullen FT. Assessing macro-level predictors and theories of crime: a meta-analysis. Crime Justice. 2005;32:373–450.

[44] Inter-university Consortium for Political and Social Research. County characteristics, 2000–2007. Ann Arbor (MI): Inter-university Consortium for Political and Social Research; 2008. 10.1111/j.1360-0443.2011.03564.x PMC4627598 21815931

[45] UCLA Institute for Digital Research and Education. Zero-inflated negative binomial regression: SAS data analysis examples. Los Angeles: UCLA Institute for Digital Research and Education; 2019 [cited 2019 Jun 1]. https://stats.idre.ucla.edu/sas/dae/zero-inflated-negative-binomial-regression.

[46] Vuong Q. Likelihood ratio tests for model selection and non-nested hypotheses. Econometrica. 1989;57:307–34.

[47] Clarke KA. A simple distribution-free test for non-nested model selection. Polit Anal. 2007;15(3):347–63.

[48] Pan W. Akaike’s information criterion in generalized estimating equations. Biometrics. 2001;57(1):120–5. 10.1111/j.0006-341x.2001.00120.x 11252586

[49] Ringle CM, Wende S, Becker JM. SmartPLS 3. Boenningstedt: SmartPLS; 2015.

[50] Baron RM, Kenny DA. The moderator–mediator variable distinction in social psychological research: conceptual, strategic, and statistical considerations. J Personality Soc Psychol. 1986;51(6):1173. 10.1037//0022-3514.51.6.1173 3806354

[51] Valeri L, VanderWeele TJ. Mediation analysis allowing for exposure–mediator interactions and causal interpretation: theoretical assumptions and implementation with SAS and SPSS macros. Psychol Methods. 2013;18(2):137 10.1037/a0031034 23379553 PMC3659198

[52] Krouse WJ, Richardson DJ. Mass murder with firearms: incidents and victims, 1999–2013. R44126. Washington (DC): Congressional Research Service; 2015.

[53] Cameron AC, Miller DL. A practitioner’s guide to cluster-robust inference. J Hum Resour. 2015;50(2):317–72.

[54] Kim D, Subramanian SV, Kawachi I. Social capital and physical health: a systematic review of the literature In: Kawachi I, Subramanian S, Kim D, editors. Social capital and health. New York: Springer; 2008 pp. 139–90.

[55] Gilbert KL, Quinn SC, Goodman RM, Butler J, Wallace J. A meta-analysis of social capital and health: a case for needed research. J Health Psychol. 2013;18(11):1385–99. 10.1177/1359105311435983 23548810 PMC4236001

[56] Sampson RJ, Raudenbush SW, Earls F. Neighborhoods and violent crime: a multilevel study of collective efficacy. Science. 1997;277(5328):918–24. 10.1126/science.277.5328.918 9252316

[57] Rosenfeld R. Documenting and explaining the 2015 homicide rise: research directions. Washington (DC): National Institute of Justice; 2016 Jun [cited 2019 Jun 1]. https://www.ncjrs.gov/pdffiles1/nij/249895.pdf.

[58] Roth R. American homicide. Cambridge (MA): Harvard University Press; 2012.

[59] Tyler Tom. R. Why people obey the law. Princeton (NJ): Princeton University Press; 2006.

[60] Kondo N, Sembajwe G, Kawachi I, van Dam RM, Subramanian SV, Yamagata Z. Income inequality, mortality, and self rated health: meta-analysis of multilevel studies. BMJ. 2009;339:b4471 10.1136/bmj.b4471 19903981 PMC2776131

[61] Roberts A, Willits D. Income inequality and homicide in the United States: consistency across different income inequality measures and disaggregated homicide types. Homicide Stud. 2015;19(1):28–57.

[62] Kramer MR, Hogue CR. Is segregation bad for your health? Epidemiol Rev. 2009;31(1):178–94. 19465747 10.1093/epirev/mxp001 PMC4362512

[63] Mehra R, Boyd LM, Ickovics JR. Racial residential segregation and adverse birth outcomes: a systematic review and meta-analysis. Soc Sci Med. 2017;191:237–50. 10.1016/j.socscimed.2017.09.018 28942206

[64] Venkataramani AS, Chatterjee P, Kawachi I, Tsai AC. Economic opportunity, health behaviors, and mortality in the United States. Am J Public Health. 2016;106(3):478–84. 10.2105/AJPH.2015.302941 26691108 PMC4758869

[65] Grossman M. On the concept of health capital and the demand for health. J Polit Econ. 1972;80:223–55.

[66] Snyder CR, Irving LM, Anderson JR. Hope and health In: Snyder CR, Forsyth DR, editors. Handbook of social and clinical psychology: the health perspective. Elmsford (NY): Pergamon Press; 1991.

[67] Dabla-Norris ME, Kochhar MK, Suphaphiphat MN, Ricka MF, Tsounta E. Causes and consequences of income inequality: a global perspective. Washington (DC): International Monetary Fund; 2015 [cited 2019 Nov 13]. https://www.imf.org/external/pubs/ft/sdn/2015/sdn1513.pdf.

[68] Rosenfeld R, Baumer E, Messner SF. Social trust, firearm prevalence, and homicide. Ann Epidemiol. 2007;17(2):119–25. 10.1016/j.annepidem.2006.07.016 17178232

[69] Kennedy BP, Kawachi I, Prothrow-Stith D, Lochner K, Gupta V. Social capital, income inequality, and firearm violent crime. Soc Sci Med. 1998;47(1):7–17. 10.1016/s0277-9536(98)00097-5 9683374

[70] Rosenfeld R, Messner SF, Baumer EP. Social capital and homicide. Soc Forces. 2001;80(1):283–310.

[71] De Coster S, Heimer K, Wittrock SM. Neighborhood disadvantage, social capital, street context, and youth violence. Sociol Q. 2006;47(4):723–53.

[72] Deller SC, Deller MA. Rural crime and social capital. Growth Change. 2010;41(2):221–75.

[73] Roth R. How the erosion of trust leads to murders and mass shootings. The Washington Post. 2017 Oct 6 [cited 2019 Jun 1]. https://www.washingtonpost.com/outlook/how-the-erosion-of-trust-leads-to-murders-and-mass-shootings/2017/10/06/382cc4b2-a91e-11e7-92d1-58c702d2d975_story.html.

[74] McCall PL, Land KC, Parker KF. Heterogeneity in the rise and decline of city-level homicide rates, 1976–2005: a latent trajectory analysis. Soc Sci Res. 2011;40(1):363–78.

[75] Messner SF, Tardiff K. Economic inequality and levels of homicide: an analysis of urban neighborhoods. Criminology. 1986;24:297–317.

[76] Kposowa AJ, Breault KD, Harrison BM. Reassessing the structural covariates of violent and property crimes in the USA: a county-level analysis. Br J Sociol. 1995;46(1):79–105.

[77] Gutierrez Rufrancos H, Power M, Pickett KE, Wilkinson R. Income inequality and crime: a review and explanation of the time-series evidence. Sociol Criminol Open Access. 2013;1(1):e103.

[78] Peterson RD, Krivo LJ. Racial segregation, the concentration of disadvantage, and black and white homicide victimization. Sociol Forum. 1999;14(3):465–93.

[79] Lee MR, Ousey GC. Counterbalancing disadvantage? Residential integration and urban black homicide. Soc Prob. 2007;54(2):240–62.

[80] Krivo LJ, Peterson RD, Kuhl DC. Segregation, racial structure, and neighborhood violent crime. Am J Sociol. 2009;114(6):1765–802. 10.1086/597285 19852253

[81] Anopov A, Rothman EF, Cronin SW, Franklin L, Cansever A, Potter F, et al The role of racial residential segregation in black-white disparities in firearm homicide at the state level in the United States, 1991–2015. J Natl Med Assoc. 2019;111(1):62–75. 10.1016/j.jnma.2018.06.002 30129481

[82] Burton CE. Segregation and Latino homicide victimization. Am J Crim Justice. 2004;29(1):21–36.

[83] Phillips JA. White, black, and Latino homicide rates: why the difference? Soc Prob. 2002;49(3):349–73.

[84] Xie M. The effects of multiple dimensions of residential segregation on black and Hispanic homicide victimization. J Quant Criminol. 2010;26(2):237–68.

[85] Tuttle J. Specifying the effect of social welfare expenditures on homicide and suicide: across-national, longitudinal examination of the stream analogy of lethal violence. Justice Q. 2018;35(1):87–113.

[86] Wilson M, Daly M. Life expectancy, economic inequality, homicide, and reproductive timing in Chicago neighborhoods. BMJ. 1997;314(7089):1271 10.1136/bmj.314.7089.1271 9154035 PMC2126620

[87] LaFree G. Losing legitimacy: street crime and the decline of social institutions in America. London: Routledge; 2018.

[88] Mabry PL, Marcus SE, Clark PI, Leischow SJ, Méndez D. Systems science: a revolution in public health policy research. Am J Public Health. 2010;100(7):1161–3. 10.2105/AJPH.2010.198176 20530757 PMC2882409

[89] The Washington Post. 994 people shot dead by police in 2015. The Washington Post; 2019 [cited 2019 Jun 1]. https://www.washingtonpost.com/graphics/national/police-shootings/.

[90] Feld S, Bauldry S. Separate, unequal, and uncorrelated: why we need to consider race-specific homicide rates in U.S. metropolitan areas. Socius. 2018;4:1–8. 10.1177/2378023118773959

[91] Greenberg D. The end of neutrality. Politico Magazine. 2018 Sep 6 [cited 2019 Jun 1]. https://www.politico.com/magazine/story/2018/09/06/common-ground-good-america-society-219616.

[92] Kim D. Projected impacts of federal tax policy proposals on mortality burden in the United States: a microsimulation analysis. Prev Med. 2018;111:272–9. 10.1016/j.ypmed.2017.10.021 29066374 PMC5911242

[93] Greenstone M, Looney A, Patashnik J, Yu M. Thirteen economic facts about social mobility and the role of education. Washington (DC): Hamilton Project; 2013.

[94] Blanden J, Gregg P, Machin S. Intergenerational mobility in Europe and North America. A report supported by the Sutton Trust. London: Centre for Economic Performance; 2005 Apr [cited 2019 Jun 1]. https://www.suttontrust.com/wp-content/uploads/2005/04/IntergenerationalMobility.pdf.

[95] Boston Consulting Group, Sutton Trust. The state of social mobility in the UK. London: Sutton Trust; 2017 Jul [cited 2019 Jun 1]. https://www.suttontrust.com/wp-content/uploads/2017/07/BCGSocial-Mobility-report-full-version_WEB_FINAL.pdf.

[96] Pike H. Life expectancy in England and Wales has fallen by six months. BMJ. 2019;364:l1123 10.1136/bmj.l1123 30858167

[97] Shaw D. Ten charts on the rise of knife crime in England and Wales. BBC News. 2019 July 18 [cited 2019 Jun 1]. https://www.bbc.com/news/uk-42749089.

