# Supplementary material for: Social determinants of health in relation to firearm-related homicides in the United States: A nationwide multilevel cross-sectional study
Source: PLoS Med. 2019 Dec 17;16(12):e1002978. doi: 10.1371/journal.pmed.1002978 (PMC6917210; doi:10.1371/journal.pmed.1002978)
Supplement: S1 STROBE Statement — (PDF) [file pmed.1002978.s001.pdf]

S1 STROBE Statement

	Item No	Recommendation
Title and abstract	1	(*a*) Indicate the study's design with a commonly used term in the title or the abstract<br>See Title
		(*b*) Provide in the abstract an informative and balanced summary of what was done and what was found<br>See Abstract
**Introduction**		
Background/rationale	2	Explain the scientific background and rationale for the investigation being reported<br>See all of Introduction except final paragraph
Objectives	3	State specific objectives, including any prespecified hypotheses<br>See Introduction, final paragraph
**Methods**		
Study design	4	Present key elements of study design early in the paper<br>See Methods, first paragraph
Setting	5	Describe the setting, locations, and relevant dates, including periods of recruitment, exposure, follow-up, and data collection<br>See Methods, second paragraph
Participants	6	(*a*) *Cohort study*—Give the eligibility criteria, and the sources and methods of selection of participants. Describe methods of follow-up<br>*Case-control study*—Give the eligibility criteria, and the sources and methods of case ascertainment and control selection. Give the rationale for the choice of cases and controls<br>*Cross-sectional study*—Give the eligibility criteria, and the sources and methods of selection of participants<br>See Methods, second paragraph
		(*b*) *Cohort study*—For matched studies, give matching criteria and number of exposed and unexposed<br>*Case-control study*—For matched studies, give matching criteria and the number of controls per case
Variables	7	Clearly define all outcomes, exposures, predictors, potential confounders, and effect modifiers. Give diagnostic criteria, if applicable<br>See Methods subsections on Outcomes, County and commuting zone level social determinant exposures, State and local social determinant exposures, and Covariates
Data sources/ measurement	8*	For each variable of interest, give sources of data and details of methods of assessment (measurement). Describe comparability of assessment methods if there is more than one group<br>See Methods subsections on Outcomes, County and commuting zone level social determinant exposures, State and local social determinant exposures, and Covariates
Bias	9	Describe any efforts to address potential sources of bias<br>See Methods subsections on Covariates, Statistical analysis
Study size	10	Explain how the study size was arrived at

		See Methods section, first paragraph
Quantitative variables	11	Explain how quantitative variables were handled in the analyses. If applicable, describe which groupings were chosen and why<br>See Methods subsection on Statistical analysis
Statistical methods	12	(*a*) Describe all statistical methods, including those used to control for confounding<br>See Methods subsection on Statistical analysis
		(*b*) Describe any methods used to examine subgroups and interactions<br>See Methods subsection on Statistical analysis, third last paragraph for mediation analysis description
		(*c*) Explain how missing data were addressed<br>See Results section, first paragraph
		(*d*) *Cohort study*—If applicable, explain how loss to follow-up was addressed<br>*Case-control study*—If applicable, explain how matching of cases and controls was addressed<br>*Cross-sectional study*—If applicable, describe analytical methods taking account of sampling strategy<br>N/A
		(*e*) Describe any sensitivity analyses<br>See Methods subsection on Statistical analysis

Continued on next page

**Results**		
Participants	13*	(a) Report numbers of individuals at each stage of study—eg numbers potentially eligible, examined for eligibility, confirmed eligible, included in the study, completing follow-up, and analysed<br>See Results section, first paragraph
		(b) Give reasons for non-participation at each stage<br>N/A
		(c) Consider use of a flow diagram
Descriptive data	14*	(a) Give characteristics of study participants (eg demographic, clinical, social) and information on exposures and potential confounders<br>See Results section, first paragraph
		(b) Indicate number of participants with missing data for each variable of interest<br>See Results section, first paragraph
		(c) *Cohort study*—Summarise follow-up time (eg, average and total amount)
Outcome data	15*	*Cohort study*—Report numbers of outcome events or summary measures over time
		Case-control study—Report numbers in each exposure category, or summary measures of exposure
		Cross-sectional study—Report numbers of outcome events or summary measures<br>See Results section, first paragraph
Main results	16	(*a*) Give unadjusted estimates and, if applicable, confounder-adjusted estimates and their precision (eg, 95% confidence interval). Make clear which confounders were adjusted for and why they were included<br>See Results section, paragraphs 2-5
		(*b*) Report category boundaries when continuous variables were categorized<br>N/A
		(*c*) If relevant, consider translating estimates of relative risk into absolute risk for a meaningful time period<br>N/A
Other analyses	17	Report other analyses done—eg analyses of subgroups and interactions, and sensitivity analyses<br>See Results section, last 4 paragraphs
**Discussion**		
Key results	18	Summarise key results with reference to study objectives<br>See Discussion section, first paragraph
Limitations	19	Discuss limitations of the study, taking into account sources of potential bias or imprecision. Discuss both direction and magnitude of any potential bias<br>See Discussion subsection on Strengths and limitations, paragraphs 2-5
Interpretation	20	Give a cautious overall interpretation of results considering objectives, limitations, multiplicity of analyses, results from similar studies, and other relevant evidence<br>See Discussion subsection on Implications of the study, second and third paragraphs
Generalisability	21	Discuss the generalisability (external validity) of the study results<br>See Discussion subsection on Strengths and limitations, first paragraph
**Other information**		
Funding	22	Give the source of funding and the role of the funders for the present study and, if applicable, for the original study on which the present article is based<br>See Funding section

*Give information separately for cases and controls in case-control studies and, if applicable, for exposed and unexposed groups in cohort and cross-sectional studies.

Note: An Explanation and Elaboration article discusses each checklist item and gives methodological background and published examples of transparent reporting. The STROBE checklist is best used in conjunction with this article (freely available on the Web sites of PLoS Medicine at http://www.plosmedicine.org/, Annals of Internal Medicine at http://www.annals.org/, and Epidemiology at http://www.epidem.com/). Information on the STROBE Initiative is available at www.strobe-statement.org.
