# Supplementary material for: Social determinants of health in relation to firearm-related homicides in the United States: A nationwide multilevel cross-sectional study
Source: PLoS Med. 2019 Dec 17;16(12):e1002978. doi: 10.1371/journal.pmed.1002978 (PMC6917210; doi:10.1371/journal.pmed.1002978)
Supplement: S1 Table — (PDF) [file pmed.1002978.s002.pdf]

<u>S1 Table</u>. Descriptive characteristics of key social determinants examined and the total number of firearm-related homicide incidents at the CT level, 2015.

Social Determinant	Mean	Standard Deviation	Range	Skewness	Kurtosis
**State and local level**					
Welfare spending per capita ($)	900.69	278.84	475, 1559	0.57	-0.77
Education spending per capita ($)	2790.20	430.81	2041, 4141	0.79	1.55
Protection spending per capita ($)	640.67	150.92	434, 1048	0.74	-0.10
**CZ level**					
Racial segregation index	0.13	0.10	0.00, 0.55	1.22	1.67
Income segregation index	0.04	0.03	0.00, 0.14	0.98	0.15
**County level**					
Gini coefficient	0.38	0.08	0.16, 0.82	0.73	1.07
Community social capital index	0.00	1.00	-1.67, 7.07	1.55	4.03
Institutional social capital index	0.00	1.00	-4.66, 2.99	-0.19	0.36
Social mobility index	43.44	5.45	23.7, 63.8	0.53	0.33
**CT level**					
Total number of firearm-related homicide incidents	0.16	0.50	0, 17	4.98	48.97

CT, census tract; CZ, commuting zone.
