# Supplementary material for: Social determinants of health in relation to firearm-related homicides in the United States: A nationwide multilevel cross-sectional study
Source: PLoS Med. 2019 Dec 17;16(12):e1002978. doi: 10.1371/journal.pmed.1002978 (PMC6917210; doi:10.1371/journal.pmed.1002978)
Supplement: S2 Table — (PDF) [file pmed.1002978.s003.pdf]

S2 Table. Social determinants, data sources, average lag periods in prior studies, and temporal order for examining mediation in present study.

Social Determinant	Data Sources	Data Year	Average Lag Period for Homicides in 2015	Average Lag Period in Longitudinal/Lagged Cross-sectional US Studies of Homicides/ External Injuries	Comments	Presence of Correct Temporal Order for Examining Mediation
**County level**						
Income inequality	1) American Community Survey (ACS) [19]<br><br>2) Internal Revenue Service Statistics of Income Sample [20]	1) 2006-2010 (5-y average, centered in 2008); 2010-2014 (5-y average, centered in 2012)<br><br>2) 1996-2000	1) 3 y,<br>7 y<br><br>2) 17 y	6-7 y (multilevel study of state income inequality and external injury mortality) [21]<br><br>5-15 y range (ecological study of county income inequality and homicide rates) [22]<br><br>10-20 y range (ecological study of state income inequality and homicide rates) [23]	1) Earliest year of ACS data available<br><br>2) Earlier data available based on income tax records	-
Social capital	Census, Current Population Survey [24-28]	2008-2015	3-4 y	No known longitudinal/lagged studies	-	Yes, as mediator of social spending
Social mobility	Internal Revenue Service Statistics of Income Sample [20]	2010-2012	4 years	No known longitudinal/lagged studies	Only data source available	Yes, as mediator of income inequality
**CZ level**						
Racial and income segregation	Census, Chetty et al. [11]	2000	15 years	No known longitudinal/lagged studies		No, for income segregation as mediator of racial segregation (contemporaneous)

**State and local level**						
Social spending	Annual Surveys of State and Local Government Finances [29]	2005, 2008, 2010	5 y<br>7 y<br>10 y	7-8 y (multilevel study of state welfare and education spending and external injury mortality) [21]<br>1 y (ecological study of state social spending and homicide rates) [30]	-	-
**CT level**						
Percent unemployed, on cash assistance, in poverty, and males living alone	American Community Survey (ACS) [19]	2010-2014 (5-y average, centered in 2012)	3 y	Examination of 1y to 7 y lags for *county* unemployment rate: strongest between-county effect on homicide rate using 3-6 y lags; strongest within-county effect using 4-5 y lags [31]<br><br>4 y (ecological study of neighborhood concentrated socioeconomic disadvantage, based on unemployment rate, poverty rate, female-headed households, and households with public assistance, and homicide rates) [32]<br><br>5-23 y follow-up range (experimental study of neighborhood relocation and homicide – association found for % with college education but not % in poverty) [33]	-	-

Evidence of the impacts of income inequality on other health outcomes suggest relatively longer lag periods; by analogy, a longer latency period was hence hypothesized and modeled here for income inequality. Other area-level social determinants were hypothesized for the present study to have lag periods intermediate in length between 3 years and 10 years. Therefore, at one extreme, factors at the smallest and most proximal area level, the CT level, were based on a 3-year lag (using 2000-2014 American Community Survey data, centered in 2012). Measures of county-level income inequality were selected using 2010-2014 American Community Survey data centered in 2012 and 2006-2010 American Community Survey data centered in 2008, for average lag periods of 3 years and 7 years, respectively; and using Internal Revenue Service Statistics of Income Sample data in 1996-2000 (centered in 1998) as published by Chetty et al. [11], for an average lag period of 17 years. State and local spending on welfare, education, protection, and total spending corresponded to the years 2005, 2008, and 2010 (for 10-, 7-, and 5-year lags, respectively). Data on residential and income segregation were available based on the 2000 Census, for a lag period of 15 years. Such a longer lag period is possible due to the downstream plausible effects of segregation on social mobility [11], a process which may take years.

In selecting the years of data for social determinant exposure variables, an attempt was also made to maintain the correct temporal order for mediation analysis where possible. Building on previous theoretical and empirical work to suggest that income inequality is a driver of social mobility [34,35], the modeled lag period for social mobility (4 years) was shorter than the final lag period selected for income inequality (17 years). Likewise, building on previous work that suggests that the welfare state can generate social capital [36,37], the modeled average lag period for social capital (3-4 years) was shorter than the final lag period selected for social spending (5 years).

CT, census tract; CZ, commuting zone.
