# Supplementary material for: Social determinants of health in relation to firearm-related homicides in the United States: A nationwide multilevel cross-sectional study
Source: PLoS Med. 2019 Dec 17;16(12):e1002978. doi: 10.1371/journal.pmed.1002978 (PMC6917210; doi:10.1371/journal.pmed.1002978)
Supplement: S4 Table — (PDF) [file pmed.1002978.s005.pdf]

S4 Table. Results from analysis of varying lag periods for state and local welfare and education spending (per $250 per capita increase) and risk of firearm-related homicide and total number of firearm-related homicide incidents at the CT level in the contiguous US, 2015.

	Lag Period Used		
	**5 years (preferred lag)**	**7 years**	**10 years**
**Table 1, Model 1 (CT gun homicide rates)**	**IRR (95% CI) *p*-Value**	**IRR (95% CI) *p*-Value**	**IRR (95% CI) *p*-Value**
Welfare spending	0.88 (0.84, 0.91) $p < 0.001$	0.75 (0.65, 0.87) $p < 0.001$	1.15 (0.92, 1.43) $p = 0.22$
Education spending	0.87 (0.76, 0.99) $p = 0.03$	0.64 (0.39, 1.05) $p = 0.08$	0.58 (0.41, 0.83) $p = 0.003$
**Table 2, Model 1 (CT total no. of gun homicide incidents)**			
Welfare spending	0.85 (0.81, 0.89) $p < 0.001$	0.84 (0.71, 0.995) $p = 0.04$	0.90 (0.73, 1.12) $p = 0.35$
Education spending	0.98 (0.85, 1.14) $p = 0.80$	1.24 (0.72, 2.13) $p = 0.44$	0.81 (0.57, 1.14) $p = 0.23$

IRR (95% CI) and *p*-values are derived from multivariate-adjusted negative binomial regression models and correspond to a 1-SD change. All models are adjusted for state fixed effects, total state and local spending, and state gun control policy indicators for concealed carry weapon carry laws, requirements for gun dealers to report records to the state, and state background check laws. At the CZ level, all models are adjusted for median household income, percentage Black, and an indicator variable for whether the CZ corresponded to an urban area. At the county level, all models are adjusted for median household income, percentage black, population density, and property crime rate. At the CT level, all models are adjusted for median household income, (median household income)^2, percentage with high school education, (percentage with high school education)^2, percentage black, (percentage black)^2, percentage male, percentage age 20-34 years, (percentage age 20-34 years)^2, total population in the year 2012, and (total population in the year 2012)^2. CI, confidence interval; CT, census tract; CZ, commuting zone; IRR, incidence rate ratio.
