# Supplementary material for: Social determinants of health in relation to firearm-related homicides in the United States: A nationwide multilevel cross-sectional study
Source: PLoS Med. 2019 Dec 17;16(12):e1002978. doi: 10.1371/journal.pmed.1002978 (PMC6917210; doi:10.1371/journal.pmed.1002978)
Supplement: S5 Table — (PDF) [file pmed.1002978.s006.pdf]

**S5 Table. Variance inflation factors for social determinant exposure variables.**

| Social Determinant | Variance Inflation Factor (VIF) |
|---|---|
| **State and local level** | |
| Welfare spending | 4.00 |
| Education spending | 3.25 |
| Protection spending | 3.51 |
| **Commuting zone level** | |
| Racial segregation | 2.64 |
| Income segregation | 3.92 |
| % Urban | 1.64 |
| % Black | 4.13 |
| Median household income | 3.15 |
| **County level** | |
| Community social capital | 2.17 |
| Institutional social capital | 2.23 |
| Social mobility | 2.59 |
| Gini coefficient | 1.90 |
| % Black | 4.90 |
| Median household income | 3.67 |
