# Supplementary material for: Social determinants of health in relation to firearm-related homicides in the United States: A nationwide multilevel cross-sectional study
Source: PLoS Med. 2019 Dec 17;16(12):e1002978. doi: 10.1371/journal.pmed.1002978 (PMC6917210; doi:10.1371/journal.pmed.1002978)
Supplement: S6 Table — (PDF) [file pmed.1002978.s007.pdf]

<u>S6 Table</u>. Coefficient estimates from generalized linear regression models exploring potential mediation pathways.*

	**Model Outcome Variable**		
	**Model A:** State and local welfare spending	**Model B:** County-level institutional social capital	**Model C:** County-level social mobility
**Exposure Variable**	**β (95% CI)** ***p*-Value**	**β (95% CI)** ***p*-Value**	**β (95% CI)** ***p*-Value**
County Gini coefficient	-0.04 (-0.07, -0.01) $p = 0.01$	-0.01 (-0.05, 0.03) $p = 0.66$	-0.08 (-0.16, -0.01) $p = 0.02$
State and local welfare spending	-	0.65 (0.60, 0.71) $p < 0.001$	-

*Model estimates correspond to one tested pathway (commonly referred to as path a) in the mediation analysis approach by Baron and Kenny [50]. For example, with the county-level Gini coefficient, state and local welfare spending, and CT-level homicide rates as the exposure, mediator, and outcome, respectively, Model A explores the Gini coefficient as a predictor of welfare spending.

All regression coefficients β (95% CI) and *p*-values are derived from multivariate-adjusted linear regression models and correspond to a 1-SD change. All models are adjusted for state fixed effects, state and local welfare (except for welfare spending as an outcome), education, protection, and total spending, and state gun control policy indicators for concealed carry weapon carry laws, requirements for gun dealers to report records to the state, and state background check laws. At the CZ level, all models are adjusted for racial and income segregation, median household income, percentage black, and an indicator variable for whether the CZ corresponded to an urban area. At the county level, all models are adjusted for community social capital, institutional social capital, social mobility, median household income, percentage black, population density, and property crime rate. At the CT level, all models are adjusted for percentage unemployed, percentage on cash assistance, percentage in poverty, percentage of males living alone, median household income, (median household income)^2, percentage with high school education, (percentage with high school education)^2, percentage Black, (percentage Black)^2, percentage male, percentage age 20-34 years, (percentage age 20-34 years)^2, total population in the year 2012, and (total population in the year 2012)^2, CI, confidence interval. CT, census tract; CZ, commuting zone.
