# Supplementary material for: Social determinants of health in relation to firearm-related homicides in the United States: A nationwide multilevel cross-sectional study
Source: PLoS Med. 2019 Dec 17;16(12):e1002978. doi: 10.1371/journal.pmed.1002978 (PMC6917210; doi:10.1371/journal.pmed.1002978)
Supplement: S8 Table — (PDF) [file pmed.1002978.s009.pdf]

S8 Table. Causal mediation analysis of welfare spending as a mediator of the association between income inequality and firearm-related homicide rate.

Simplified causal diagram for mediation analysis:

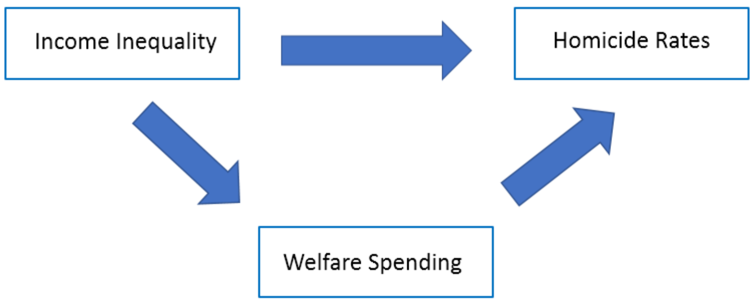

	IRR or %	95% CI	*p*-Value
**Total Effect**	1.06	1.00, 1.11	0.04
**Controlled Direct Effect**	1.06	1.00, 1.11	0.047
**Natural Direct Effect**	1.06	1.00, 1.11	0.046
**Natural Indirect Effect**	1.00	1.00, 1.01	0.32
**% Mediated**	3.17	-3.72, 10.06	0.37
**% Due to Interaction**	-0.69	-2.57, 1.19	0.47

IRR (95% CI) and *p*-values are derived from multivariate-adjusted negative binomial models and correspond to a 1-SD change. All models are adjusted for census statistical division fixed effects, state and local education, protection, and total spending, and state gun control policy indicators for concealed carry weapon carry laws, requirements for gun dealers to report records to the state, and state background check laws. At the CZ level, all models are adjusted for racial and income segregation, median household income, percentage black, and an indicator variable for whether the CZ corresponded to an urban area. At the county level, all models are adjusted for community social capital, institutional social capital, and percentage black. At the CT level, all models are adjusted for the percentage unemployed, percentage on cash assistance, percentage in poverty, percentage of males living alone, median household income, percentage with high school education, percentage black, percentage male, and percentage age 20-34 years. CI, confidence interval; CT, census tract; CZ, commuting zone; IRR, incidence rate ratio.
