# Supplementary material for: Social determinants of health in relation to firearm-related homicides in the United States: A nationwide multilevel cross-sectional study
Source: PLoS Med. 2019 Dec 17;16(12):e1002978. doi: 10.1371/journal.pmed.1002978 (PMC6917210; doi:10.1371/journal.pmed.1002978)
Supplement: S12 Table — (PDF) [file pmed.1002978.s013.pdf]

S12 Table. Social determinants and risk of firearm-related homicide at the CT level in the contiguous US, 2015.

Social Determinant	Model 1		Model 2		Model 3		Model 4	
	IRR (95% CI)	*p*-Value	IRR (95% CI)	*p*-Value	IRR (95% CI)	*p*-Value	IRR (95% CI)	*p*-Value
**County level**								
Gini coefficient (0.1 unit)	1.10 (1.01, 1.18)	0.02	1.09 (1.01, 1.17)	0.02	1.10 (1.02, 1.17)	0.01	1.07 (1.01, 1.13)	0.03
Community social capital (1 unit)	-	-	-	-	0.96 (0.86, 1.08)	0.50	0.99 (0.88, 1.10)	0.83
Institutional social capital (1 unit)	-	-	-	-	0.81 (0.73, 0.91)	<0.001	0.82 (0.74, 0.91)	<0.001
Social mobility (5 units)	-	-	-	-	-	-	0.77 (0.69, 0.87)	<0.001
**CZ level**								
Racial segregation (0.1 unit)	1.03 (0.96, 1.12)	0.39	1.02 (0.93, 1.10)	0.73	1.01 (0.92, 1.10)	0.87	1.01 (0.93, 1.10)	0.79
Income segregation (0.05 unit)	-	-	1.08 (0.92, 1.27)	0.36	1.08 (0.92, 1.27)	0.35	1.03 (0.88, 1.21)	0.67
**State and local level**								
Welfare spending ($250)	0.88 (0.84, 0.91)	<0.001	0.87 (0.84, 0.91)	<0.001	1.02 (0.93, 1.13)	0.65	1.00 (0.90, 1.10)	0.97
Education spending ($250)	0.87 (0.76, 0.99)	0.03	0.94 (0.75, 1.17)	0.57	1.04 (0.82, 1.34)	0.73	0.84 (0.67, 1.05)	0.13
Protection spending ($250)	0.90 (0.73, 1.11)	0.32	0.89 (0.72, 1.10)	0.26	0.95 (0.77, 1.18)	0.66	0.91 (0.74, 1.11)	0.33
**CT Level**								
Percent unemployed (1%)	1.01 (1.01, 1.02)	<0.001	1.01 (1.01, 1.02)	<0.001	1.01 (1.01, 1.02)	0.002	1.01 (1.00, 1.02)	0.01
Percent on cash assistance (1%)	1.03 (1.02, 1.04)	<0.001	1.03 (1.02, 1.04)	<0.001	1.03 (1.02, 1.04)	<0.001	1.03 (1.02, 1.04)	<0.001
Percent in poverty (1%)	1.02 (1.01, 1.03)	<0.001	1.02 (1.01, 1.03)	<0.001	1.02 (1.01, 1.03)	<0.001	1.02 (1.01, 1.03)	<0.001
(Percent in poverty)^2 (1%)	0.9998 (0.9997, 0.9999)	<0.001	0.9998 (0.9997, 0.9999)	<0.001	0.9998 (0.9997, 0.9999)	<0.001	0.9998 (0.9997, 0.9999)	<0.001
Percent males living alone (1%)	1.03 (1.02, 1.04)	<0.001	1.03 (1.02, 1.04)	<0.001	1.03 (1.02, 1.04)	<0.001	1.03 (1.02, 1.04)	<0.001

IRR (95% CI) and *p*-values are derived from multivariate-adjusted negative binomial regression models and correspond to a unit change as shown above in parentheses. All models are adjusted for state fixed effects, total state and local spending, and state gun control policy indicators for concealed carry weapon carry laws, requirements for gun dealers to report records to the state, and state background check laws. At the CZ level, all models are adjusted for median household income, percentage black, and an indicator variable for whether the CZ corresponded to an urban area. At the county level, all models are adjusted for median household income, percentage black, population density, and property crime rate. At the CT level, all models are adjusted for median household income, (median household income)^2, percentage with high school education, (percentage with high school education)^2, percentage black, (percentage black)^2, percentage male, percentage age 20-34 years, (percentage age 20-34 years)^2, total population in the year 2012, and (total population in the year 2012)^2. CI, confidence interval; CT, census tract; CZ, commuting zone; IRR, incidence rate ratio.
